# Intermediate field directions recorded in Pliocene basalts in Styria (Austria): evidence for cryptochron C2r.2r-1

**DOI:** 10.1186/s40623-021-01518-w

**Published:** 2021-10-03

**Authors:** Elisabeth Schnepp, Patrick Arneitz, Morgan Ganerød, Robert Scholger, Ingomar Fritz, Ramon Egli, Roman Leonhardt

**Affiliations:** 1 Palaeomagnetic Laboratory Gams, Chair of Applied Geophysics, Montanuniversität Leoben, Gams 45, 8130 Frohnleiten, Austria; 2 Conrad Observatorium, ZAMG-Zentralanstalt für Meteorologie und Geodynamik, Hohe Warte 38, 1190 Vienna, Austria; 3 Geological Survey of Norway, Torgarden, P.O. Box 6315, 7491 Trondheim, Norway; 4 Universalmuseum Joanneum, Studienzentrum Naturkunde, Weinzöttlstraße 16, 8045 Graz, Austria

**Keywords:** Paleomagnetism, Paleointensity, Transitional field configuration, Cryptochron C2r.2r-1, ^39^Ar/^40^Ar dating, Styria (Austria), Volcanic rocks

## Abstract

Pliocene volcanic rocks from south-east Austria were paleomagnetically investigated. Samples were taken from 28 sites located on eight different volcanoes. Rock magnetic investigations revealed that magnetic carriers are Ti-rich or Ti-poor titanomagnetites with mainly pseudo-single-domain characteristics. Characteristic remanent magnetization directions were obtained from alternating field as well as from thermal demagnetization. Four localities give reversed directions agreeing with the expected direction from secular variation. Another four localities of the Klöch–Königsberg volcanic complex (3) and the Neuhaus volcano (1) have reversed directions with shallow inclinations and declinations of about 240° while the locality Steinberg yields a positive inclination of about 30° and 200° declination. These aberrant directions cannot be explained by local or regional tectonic movements. All virtual geomagnetic pole positions are located on the southern hemisphere. Four virtual geomagnetic poles lie close to the geographic pole, while all others are concentrated in a narrow longitude sector offshore South America (310°–355°) with low virtual geomagnetic pole latitudes ranging from − 15° to − 70°. The hypothesis that a transitional geomagnetic field configuration was recorded during the short volcanic activity of these five localities is supported by 9 paleointensity results and ^39^Ar/^40^Ar dating. Virtual geomagnetic dipole moments range from 1.1 to 2.9·10^22^ Am^2^ for sites with low VGP latitudes below about 60° and from 3.0 to 9.3·10^22^ Am^2^ for sites with higher virtual geomagnetic pole latitudes. The new ^39^Ar/^40^Ar ages of 2.51 ± 0.27 Ma for Klöch and 2.39 ± 0.03 Ma for Steinberg allow for the correlation of the Styrian transitional directions with cryptochron C2r.2r-1 of the geomagnetic polarity time scale.

**Graphic abstract:**

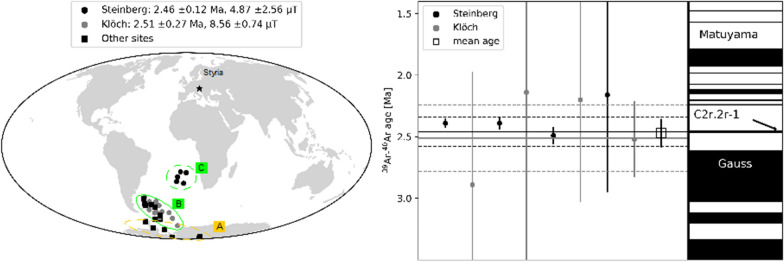

**Supplementary Information:**

The online version contains supplementary material available at 10.1186/s40623-021-01518-w.

## Introduction

The Earth’s magnetic field is generated in the outer core by an interacting system of liquid and electrical currents, which form the so-called geodynamo. For our understanding of the geodynamo knowledge of the temporal change of the geomagnetic field is crucial. Direct observations cover only a few hundred years (e.g., Arneitz et al. 2017). Paleomagnetic records obtained from rocks provide information on the geological past (e.g., Gubbins and Herrero-Bervera 2007). Volcanic rocks give spot readings of the geomagnetic field and eventually provide a temporal succession if stratigraphy is known. In addition to the stable field configuration and its secular variation, documenting transitional field configurations during reversals or excursions are of high interest in order to constrain geodynamo mechanisms and support modeling of the geomagnetic field evolution during such critical events (e.g., Leonhardt et al. 2009).

The geomagnetic polarity time scale (GPTS, e.g., Gradstein et al. 2012) is continually refined (Ogg et al. 2016). It is based on long sedimentary series obtained from ocean drilling and on records from lavas which are precisely correlated by astrochronology and radiometric dating (e.g., Channell et al. 2009; Singer 2014). The last 5 Ma of the GPTS is divided into chrons and subchrons by more than 20 polarity transitions. Further instabilities, called cryptochrons or excursions, such as short-living or aborted reversals, are documented (e.g., Singer 2014).

Here, we present a re-investigation of sites from the Styrian volcanic field in south-east Austria for which paleomagnetic directions have been published some decades ago (Pohl and Soffel 1982; Mauritsch 1972; Table 1). While the Miocene sites showed normal directions in agreement with secular variation, the Pliocene sites (Bojar et al. 2013) yielded mainly inverse directions which do not lie within the range expected from secular variation. These anomalous directions observed in Styria potentially recorded excursional or transitional states of the geomagnetic field. In order to test this hypothesis, most sites have been resampled and new sites have been added. Here, full vector paleomagnetic data obtained with state-of-the-art demagnetization and paleointensity techniques are provided for the Pliocene volcanoes. New ^39^Ar/^40^Ar dating of two sites with transitional directions improved previous K–Ar age constraints of Bojar et al. (2013) and support a correlation with the geomagnetic polarity time scale.

**Table 1:** Paleomagnetically investigated sites in the Styrian Basin

Location	Latitude (°N)	Longitude (°E)	Name	N	D (°)	I (°)	k	α_95_ (°)	Plat (°N)	PLong (°E)	dp (°)	dm (°)	δ (°)	Polarity	Reference
Altenmarkt bei Riegersburg, lava flow, Basanit, Pliocene, Fritz (1992)															
Altenmarkt	47.005	15.912	AM03	6	187.6	− 64.3	1276.0	1.9	− 84.7	293.7	2.4	3.0	3.3	R	This study
Stradner Kogel (Wilhelmsdorf), vent with dikes, Nephelinite, 1.71 ± 0.72, K/Ar, Balogh et al. (1994)															
Stradner Kogel	46.841	15.926	SK02	18	198.5	− 57.6	231.3	2.3	− 73.9	311.6	2.4	3.3	11.4	R	This study
Waltra	46.849	15.952	WA02	12	204.8	− 60.6	170.1	3.3	− 71.5	293.4	3.9	5.1	12.0	R	This study
Stradner Kogel	46.847	15.935	SK	52	200.0	− 60.0	87.9	2.1	− 62.5	301.2			10.4	R	1
Waltra	46.85	15.953	WA	10	213.0	− 58.0	34.0	7.6	− 64.6	292.3			17.0	R	2
Stein, intrusion, Basalt with Olivin, Pliocene, Balogh et al. (1994)															
Stein	46.991	16.082	ST02	11	174.7	− 61.1	289.3	2.7	− 83.9	56.2	3.2	4.1	4.6	R	This study
Stein	46.992	16.083	ST	20	176	− 64	192	2.3	− 87	82.7			2.0	R	2
Klöch-Königsberg volcanic complex Klöch Quarry, vent with dikes, Nephelinebasanite, 2.56 ± 1.2 Ma, K/Ar, Balogh et al. (1994)															
Klöch 01	46.769	15.964	KN01	7	220.0	− 30.5	96.0	6.2	− 45.2	314.9	3.8	6.9	42.3	T	This study
Klöch 02	46.769	15.964	KN02	9	217.7	– 34.5	43.9	7.9	− 48.5	315.1	5.2	9.0	37.8	T	This study
Klöch 03	46.77	15.964	KN03	6	217.6	− 32.9	101.3	6.7	− 47.8	316.3	4.3	7.6	39.2	T	This study
Klöch 04	46.77	15.965	KN04	5	202.5	− 32.7	30.9	14.0	− 55.6	335.8	9.0	15.8	35.0	T	This study
Klöch 05	46.768	15.964	KN05	7	198.6	− 39.1	18.9	14.2	− 61.2	338.2	10.2	17.0	27.9	T	This study
Klöch 06	46.767	15.965	KN06	5	207.9	− 35.7	127.3	6.8	− 54.7	326.2	4.6	7.9	33.5	T	This study
Klöch 07	46.767	15.965	KN07	5	217.2	− 41.9	78.3	8.7	− 52.7	310.3	6.5	10.7	31.1	T	This study
Klöch 08	46.768	15.964	KN08	5	214.3	− 33.5	255.1	4.8	− 50.0	319.6	3.1	5.5	37.6	T	This study
Klöch 09	46.768	15.964	KN09	3	192.6	− 47.2	180.5	9.2	− 69.1	343.3	7.7	11.9	18.9	(T)	This study
Klöch 11	46.773	15.968	KN11	6	215.3	− 24.6	55.6	9.1	− 45.0	323.2	5.2	9.7	46.2	T	This study
Klöch 12	46.767	15.968	KN12	9	211.6	− 40.1	69.5	6.2	− 55.2	318.3	4.5	7.5	30.7	T	This study
Klöch 13	46.768	15.967	KN13	10	207.4	− 45.6	65.8	6.0	− 60.8	318.8	4.9	7.6	24.4	(T)	This study
Seindl	46.763	15.953	SL	12	209.5	− 49.6	17.0	9.8	− 62.0	310.9			21.7	(T)	2
Klöch SW	46.763	15.963	S1	9	211.9	− 39.9	179.0	3.5	− 54.8	317.8			31.0	T	2
Klöch N	46.767	15.967	S2	39	224.4	− 28.6	25.0	4.5	− 41.5	311.1			45.6	T	2
Klöch 01-13	46.769	15.965	KN01-13	83	211.5	− 37.2	39.8	2.5	− 53.6	320.6	1.7	2.9		T	This study
Klöch Quarry, synvolcanic lake sediment with several layers of accretionary lapilli, reddish, sandy clay, Pliocene															
Klöch 10	46.775	15.963	KN10	6	212.5	− 42.3	159.3	5.3	− 55.8	315.4	4.0	6.6	29.0	T	This study
Zaraberg, baked tuff with lava flow, Nephelinebasanite, Pliocene, Balogh et al. (1994)															
Klöch 14	46.764	15.946	KN14	8	211.8	-28.8	95.3	5.7	− 48.9	325.2	3.5	6.3	41.2	T	This study
Klöch 15	46.764	15.946	KN15	5	219.2	− 33.5	403.2	3.8	− 47.1	314.1	2.5	4.3	39.2	T	This study
Klöch 1415	46.764	15.946	KN14-15	13	214.6	− 30.6	106.9	4.0	− 48.4	320.9	2.5	4.5		T	this study
Reichel	46.773	15.952	RL	17	222.0	− 37.3	123.0	3.1	− 47.3	308.3			36.9	T	2
Königsberg, tuff with dikes, 2.17 ± 0.13 Ma, K/Ar, Seghedi et al. (2004)															
Tieschen 01	46.783	15.954	TD01	13	214.4	− 33.7	85.3	4.5	− 50.1	319.4	2.9	5.1	37.4	T	This study
Tieschen 02	46.785	15.956	TD02	9	221.2	− 39.4	275.4	3.1	− 48.9	308.1	2.2	3.7	34.7	T	This study
Tieschen 03	46.787	15.956	TS03/TD04	17	207.9	− 42.2	71.1	4.3	− 58.4	321.4	3.2	5.2	27.6	T	This study
Neuhaus, intrusion, Basalt with Olivin, 3.76 ± 0.41 Ma and 3.11 ± 0.75 Ma, K/Ar, Balogh et al. (1994)															
Neuhaus	46.865	16.027	NH02	7	217.8	−33.4	69.2	7.3	−47.8	315.8	4.7	8.3	38.9	T	This study
Neuhaus	46.867	16.028	NH	9	224.5	− 31.8	183.0	3.4	− 43.0	309.2			42.7	T	2
Steinberg (Mühldorf), vent with dikes, Olivin nephelinite, 2.64 ± 0.55 Ma, 3.05 ± 1.4 Ma, 2.30 ± 0.14 Ma, K/Ar, Balogh et al. (1994)															
Steinberg 01	46.935	15.917	SB01	9	196.8	22.1	15.5	13.5	− 29.7	356.9	7.6	14.3	88.0	T	This study
Steinberg 02	46.935	15.917	SB02	9	201.9	35.9	79.9	5.8	− 20.3	353.9	3.9	6.7	102.3	T	This study
Steinberg 03	46.934	15.916	SB03	10	203.6	21.7	11.3	15.0	− 28.1	349.5	8.4	15.9	88.5	T	This study
Steinberg 04	46.935	15.918	SB04	8	196.2	36.6	52.2	7.7	− 21.1	359.6	5.3	9.0	102.3	T	This study
Steinberg 05	46.935	15.919	SB05	10	203.7	28.2	31.3	8.8	− 24.5	350.7	5.3	9.6	95.0	T	This study
Steinberg SB	46.935	15.917	SB01-05	46	200.7	28.8	20.8	4.7	− 25.0	353.8	2.9	5.2	95.1	T	This study

For each volcanic unit the name is given together with the kind of exposure, rock type, age, its standard deviation, dating method and the reference is given. For the sites, the name of location; latitude; longitude; site name; number of samples; declination and inclination of the characteristic remanent magnetization; precision parameter and 95% confidence circle radius of the Fisher (1953) statistics; latitude and longitude of the virtual magnetic pole position with half angles of the error ellipses; reversal angle (Prévot et al. 1985); polarity (N: normal, R: reversed, T: transitional); and reference (1: Mauritsch 1972; 2: Pohl and Soffel 1982) are listed

### Geological setting and field work

**Fig. 1:**
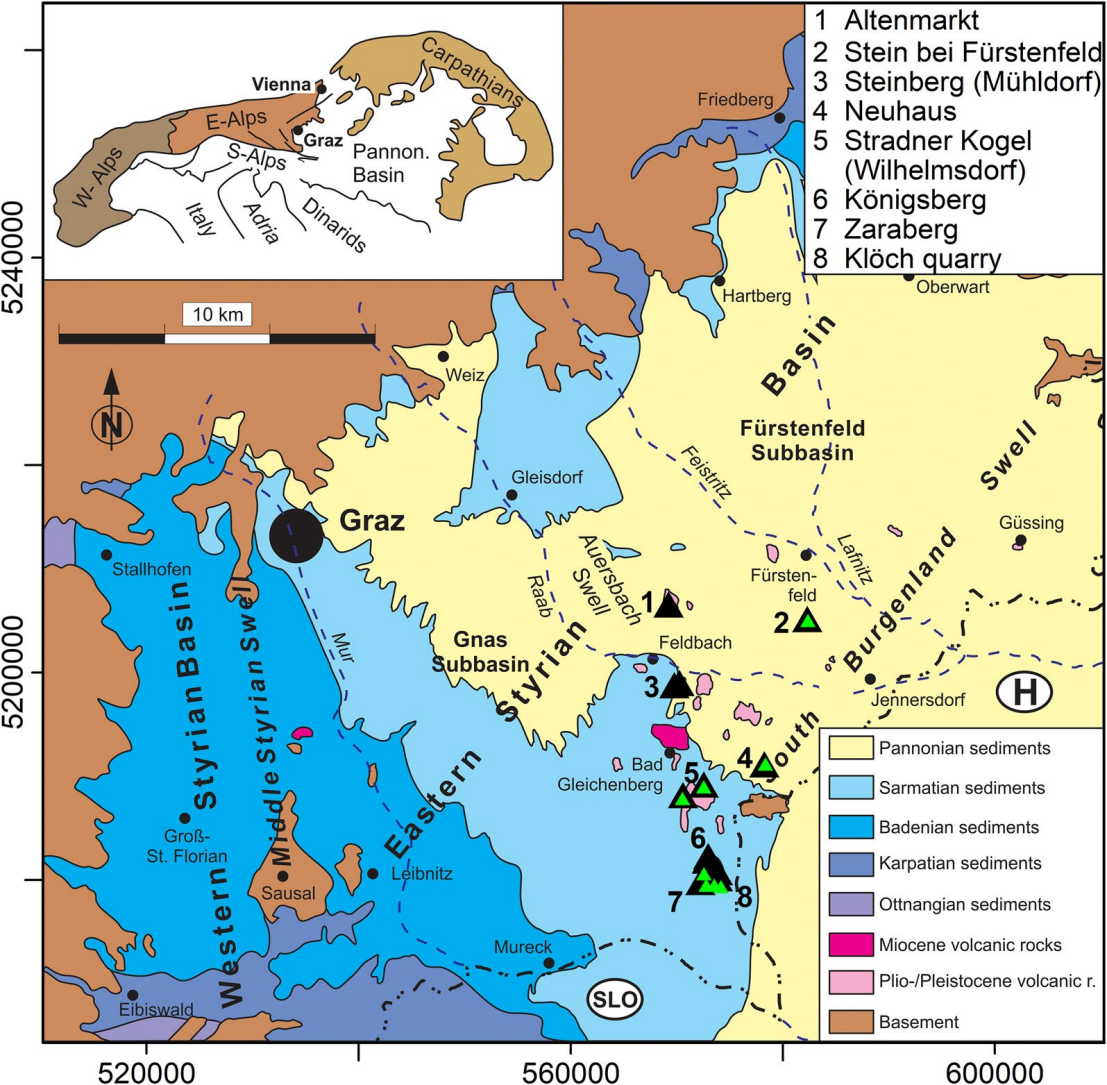
Geological setting (modified from Gross et al. 2007) and sampling locations of this study (black triangles) and of Pohl and Soffel (1982, green triangles)

The sampling area is located at the south-eastern margin of the Alps in the Styrian Basin (Fig. 1, Gross et al. 2007). It is part of the Pannonian Basin and separated from other sub-basins by the South Burgenland Swell in the south-east. The north-eastern boundary is built by a Penninic unit, while in the north, west and southwest the Styrian Basin is encircled by crystalline Austroalpine units and the Graz Paleozoic, which forms the basement as well. The formation of the Styrian Basin started in the Late Oligocene to Miocene at the final collision stage of the Adriatic and European plates and is connected to the alpidic lateral extrusion and subduction-related extension in the Pannonian region (e.g., Neubauer and Genser 1990). The sedimentary filling of the basin started in the Early Miocene and the major tectonic events were accompanied by volcanism producing volcaniclastics and high-K effusive rocks (Ebner and Sachsenhofer 1991). The second volcanic phase, starting in the Upper Miocene (Bojar et al. 2013), is dominated by a large number of tuff occurrences with phreatomagmatic origin (Pöschl 1991; Fritz 1996) and continued up to the Pliocene/Early Pleistocene with effusive alkaline rocks and volcaniclastic tuffs (Balogh et al. 1994). A total of 30–40 eruption centers have been identified (Kollmann 1965). The different volcaniclastic rocks prove the former presence of maars, scoria cones, lava lakes and lava flows. Today, exposed volcanic remnants (e.g., lava flows, diatremes) form prominent highs in the landscape (Gross et al. 2007). K–Ar ages and petrological description are available for six Pliocene lavas (see Table 1; Seghedi et al. 2004; Balogh et al. 1994).

Paleomagnetic sampling was carried out in several campaigns using an electrically or gasoline powered drill with diamond bit. The cores were orientated with a magnetic compass and whenever possible, with a sun compass. Azimuth differences between magnetic and sun compass readings were always < 5°. Samples were taken from 28 sites distributed over 8 volcanic units (see Table 1; Fig. 1). Two of the volcanic edifices have been sampled extensively: the Klöch–Königsberg complex, with samples taken in and around the big quarry of Klöch (KN01 to KN13) and at Zaraberg (KN14, KN15), and 3 dikes outcropping on the Königsberg tuff cone (Tieschen: TS, TD). Additionally, the synvolcanic lake sediment deposited on the top of the lavas in Klöch quarry was sampled with plastic boxes (Table 1). Another five sites were distributed over the big quarry Steinberg south of Mühldorf (SB01 to SB05). Directional results from six of our sites had already been published by Pohl and Soffel (1982) and were resampled at the same places or nearby.

### Rock and paleomagnetic laboratory procedures

Thermomagnetic bulk susceptibility curves of crushed material, *κ(T)*, were measured in air using a MFK1-FA susceptibility meter (Agico) with a CS-3 temperature control unit. For a better understanding of the development of thermal alteration, the effects of thermal cycling with starting temperatures between 150–290 °C and increments of 100 °C were also investigated. Continuous thermal demagnetization was performed for a few mini-specimens drilled along the characteristic remanent magnetization direction in order to check for evidence of self-reversal magnetizations. Hysteresis and isothermal remanent magnetization (IRM) backfield curves were measured on mini-cores (4–5 mm) with a Micromag Model 3900 vibration sample magnetometer (VSM).

Characteristic remanent magnetizations obtained with stepwise alternating field (AF) as well as thermal demagnetization (except site KN10) have been measured with a cryogenic (2G) or a spinner (Agico JR6) magnetometer. AF demagnetization was performed in line with the magnetometer or with external devices (MI AFD 300, Magnon International, or ASC D-2000) while a MMTD60 or a MMTDSC furnace (Magnetic measurements) was used for thermal demagnetization. AF demagnetization was performed in 10–18 steps up to a maximum field of 140–300 mT depending on the behavior of the specimen and the equipment. Thermal demagnetization was based on 10–13 steps between 100 and 600 °C with increments of 20, 40 or 50 °C. The experiment was stopped when the residual magnetization was lower than 5% of the initial natural remanent magnetization (NRM) or an increase of magnetization was observed above 400 °C.

Selection of well-suited specimens for paleointensity determination was difficult because rock magnetic experiments often revealed the presence of magnetic carriers with low thermal stability. Furthermore, viscous overprints, which hamper paleointensity determination have been detected in many samples. In addition to thermal stability of *κ(T)*-cycling and lack of viscous overprints, specimens for paleointensity determination were selected according to the absence or weakness of secondary components and remanence stability during thermal demagnetization. Temperature steps were adjusted to the expected unblocking temperatures and the onset of thermal alteration. Paleointensity experiments were conducted on 70 specimens (18 standard inch specimens and 52 mini-specimens of 9 mm diameter drilled from the inch specimens) from 14 sites (Additional file 1: Table S1). Thirteen sites were not tested because they failed the selection criteria given above. The Thellier technique modified by Coe (1967) using the protocol MT4 of Leonhardt et al. (2004) was applied. Thirteen to 15 heating steps starting at 90 °C or 200 °C, depending on the expected blocking temperature range, have been used. Temperature increments comprised between 20 and 50 °C and the maximum temperature was 500 °C, 550 °C or ~ 600 °C. A laboratory field between 15 and 40 µT adjusted to the expected intensity was applied. Six or 7 pTRM-checks, 5 or 6 tail checks (Riisager and Riisager 2001) and 3–5 additivity checks (Krása et al. 2003) were included. Heating was performed in a MMTD24 furnace (magnetic measurements) using the rapid cooling rate setting, magnetization was measured with an AGICO JR-6A Dual Spinner Magnetometer and susceptibility was checked with a Bartington MS2B/MS3 Magnetic Susceptibility Meter ASC D-2000 after each temperature step. Because chemical alteration sometimes started before the secondary component was removed, Thellier experiments were combined with AF demagnetization for 42 specimens (Marshall et al. 1988) by applying a 15-mT AF demagnetization prior to each magnetization measurement (Additional file 1: Table S1). All paleointensity measurements were carried out at the Conrad Observatory (ZAMG).

### Isotopic dating

Specimens from 4 of our sites were selected for ^39^Ar/^40^Ar-dating, but inspection of thin sections revealed that only 2 sites provided material suited for dating.

The samples were crushed and sieved to obtain 180–250 μm fractions. The finer particles were decanted in tap water and the coarser residue ultrasonically washed in acetone and deionized water several times. The optically best suited grains, void of any coatings, were handpicked under a stereomicroscope. The samples were packed in aluminum capsules together with the Taylor Creek Rhyolite (TCR) flux monitor standard along with zero age reagent grade K_2_SO_4_ and optical grade CaF_2_ salts for interference corrections. The samples were irradiated at the MTA reactor (Hungary) for ~ 2 h, with a nominal fast neutron flux density of c. 5.5 × 10^13^ n∙cm^−2^∙s^−1^. The interference correction factors for the production of isotopes from Ca and K are reported in the Additional file 2: Table S2. The groundmass grains were placed in a 3.5-mm pit size aluminum sample disk and step heated using a defocused 3.5-mm CO_2_ laser beam from Photon Machine Fusions 10.6 with a flat energy spectrum. The gases extracted from the sample cell were expanded into a Piston Free Stirling Cryocooler for trapping potential water vapor, and further expanded into a two-stage low volume extraction line (~ 300 cm^3^). Both stages were equipped with SAES GP-50 (st101 alloy) getters, the first running hot (c. 350 °C) and the second running at room temperature. The samples were analyzed with an MAP 215-50 mass spectrometer in static mode, installed at the Geological Survey of Norway. The peaks and baseline (AMU = 36.2) were determined during peak hopping for 15 cycles (15 integrations per cycle, 30 integrations on mass ^36^Ar) on the different masses (^40–36^Ar) on a MasCom electron multiplier (MC217) in analogue mode and linearly regressed back to zero inlet time. Blanks were analyzed every third measurement. After blank correction, a correction for mass fractionation, ^37^Ar and ^39^Ar decay and neutron-induced interference reactions produced in the reactor was performed using in-house software AgeMonster, written by Ganerød. This software implements the equations of McDougall and Harrison (1999) and the newly proposed decay constant for ^40^ K after Renne et al. (2010). A^40^Ar/^36^Ar ratio of 298.56 ± 0.31 from Lee et al. (2006) was used for the atmospheric argon correction and mass discrimination calculation using a power law distribution of the masses. Finally, we calculated J-values relative to an age of 28.619 ± 0.036 Ma for the TCR fluence monitor (Renne et al. 2010).

## Results

### Rock magnetism

**Fig. 2:**
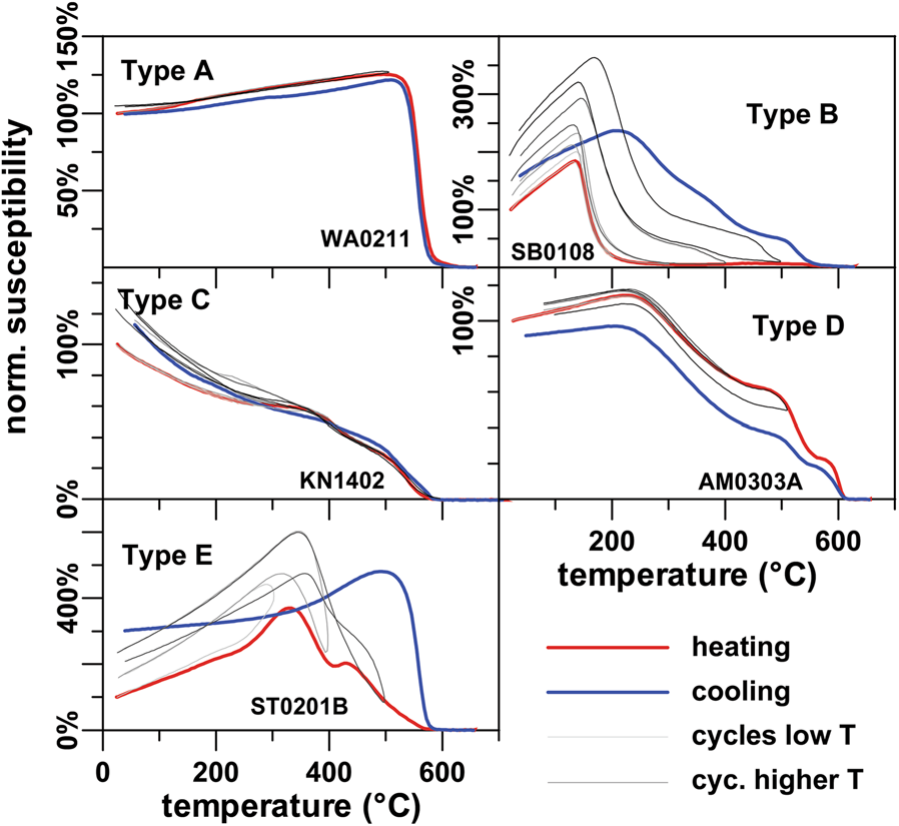
Representative examples of five types of bulk susceptibility thermomagnetic curves. Heating is shown in red, cooling in blue, and thermal cycling in gray/black

The thermomagnetic behavior of susceptibility was measured for at least one sample per site. It was studied more intensively for the sites selected for paleointensity determination using also thermal cycling runs. The investigated samples yielded a broad spectrum of *κ*(*T)*-curves. These curves can be roughly divided into five types (Fig. 2).

Type A has a single, high Curie point (*T*_c_) between 512 and 598 °C and can be almost completely reversible, which points to presence of magnetite as the dominant magnetic carrier. Irreversible curves are more common, with a susceptibility decrease to about half of the initial value (e.g., Additional file 5: Fig. S1, appendix: KN15-6). Thermal cycling shows that alteration starts above 450–500 °C. In some cases, also a second Curie point may occur at a higher temperature, pointing to the presence of some maghemite or hematite (e.g., Additional file 5: Fig. S1, KN12-7, SK0202B). Type A was found in only five localities (KN, TS/TD, SB, SK and WA, see also Additional file 5: Fig. S1, appendix and Additional file 3: Table S3).

The overwhelming majority of the sites showed a much less stable thermomagnetic behavior (Type B). It is characterized by the presence of a low Curie point, presumable carried by Ti-rich titanomagnetite, which transforms to magnetite when heated above 600 °C. Curves can be almost reversible also at higher temperatures (e.g., Additional file 5: Fig. S1, KN06-2), but normally the formation of new magnetite is indicated by a bump present below 500 °C resulting in a new Curie temperature around 580 °C (Fig. 2 and e.g., Additional file 5: Fig. S1 KN04-5, KN08-5, NH0202I, SB0106I, SB0301, SB0507I, ST0209I). Thermal cycling shows that the onset of alteration starts already around 300 °C. Heating to lower temperatures often yielded *T*_c_ values of 120–350 °C. Type B samples are thus characterized by a highly variable Ti-content. Thermal alteration often prevented a proper estimation of *T*_c_. Thellier experiments of type B specimens were performed from 90 to 360 °C in 30 °C-steps, because thermal cycling often revealed good reversibility up to 400 °C. Nevertheless, the presence of viscous overprints led to the failure of Thellier experiments for all tested type B specimens.

Type C shows a steady decrease of susceptibility with a high Curie point between 544 and 581 °C and some reversible curves were obtained. In the other cases, alteration is relatively weak, starts mostly around 500 °C (e.g., Additional file 5: Fig. S1, KN14-1O), and is visible mainly in the low (< 400 °C) temperature range. The susceptibility is relatively low and paramagnetic behavior is observed. This type is only present in one sample of SB02 (Additional file 5: Fig. S1) and site KN14, a tuff baked by the overlying lava flow KN15.

Type D shows three bumps both in heating and cooling curves, which may represent three magnetic carriers associated with Ti-rich and Ti-poor magnetite and/or maghemite. A proper *T*_c_ determination is possible only for the third decay and yields to values above 600 °C. Such curves were only found in two sites: AM03 and TS03 (cf. Additional file 5: Fig. S1).

Finally, type E shows a mixture of two or three minerals and strong alteration. A high Curie point at ~ 580 °C indicates the formation of magnetite, while all other phases disappear. Stability against thermal cycling is low and alteration starts at about 300 °C. Sites NH02 and ST02 were dominated by this behavior (cf. Additional file 5: Fig. S1).

Samples with *κ*(*T*)-types B and E were not used for paleointensity experiments, except for sites where better material was not available (cf. Additional file 3: Table S3).

**Fig. 3:**
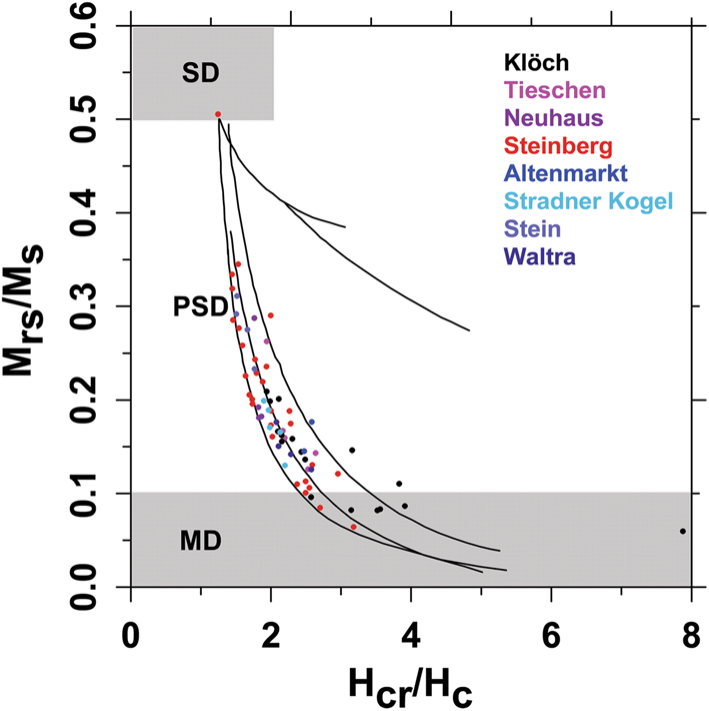
Hysteresis parameters (ratio of saturation magnetizations vs ratio of coercivity) plotted in a Day-diagram (Day et al. 1977) with fields of single domain (SD), pseudo-single domain (PSD) multi-domain (MD) grains. Black lines indicate mixtures of SD and MD grains after Dunlop (2002)

Hysteresis parameters were measured with a VSM for 2–7 per site. The Day-diagram (Fig. 3; Additional file 4: Table S4) indicates that most samples plot in the lower part of the pseudo-single domain range close to the three mixing curves of SD and MD magnetite grains of Dunlop (2002).

### Paleomagnetic directions

Directions of NRM are well constrained for only 8 sites (see Additional file 6: Fig. S2, appendix: AM03, SK02, ST02, WA02, KN01, KN12, KN14, KN15), while weak to significant secondary overprints are present at 20 sites indicated by moderate to considerable scatter of the NRM directions. Demagnetization experiments were done with AF (181 specimens) and thermally (142 specimens) and processed with the Remasoft 3.0 software in order to obtain the characteristic remanent magnetization (ChRM) directions from principal component analysis (PCA, Kirschvink 1980).

**Fig. 4:**
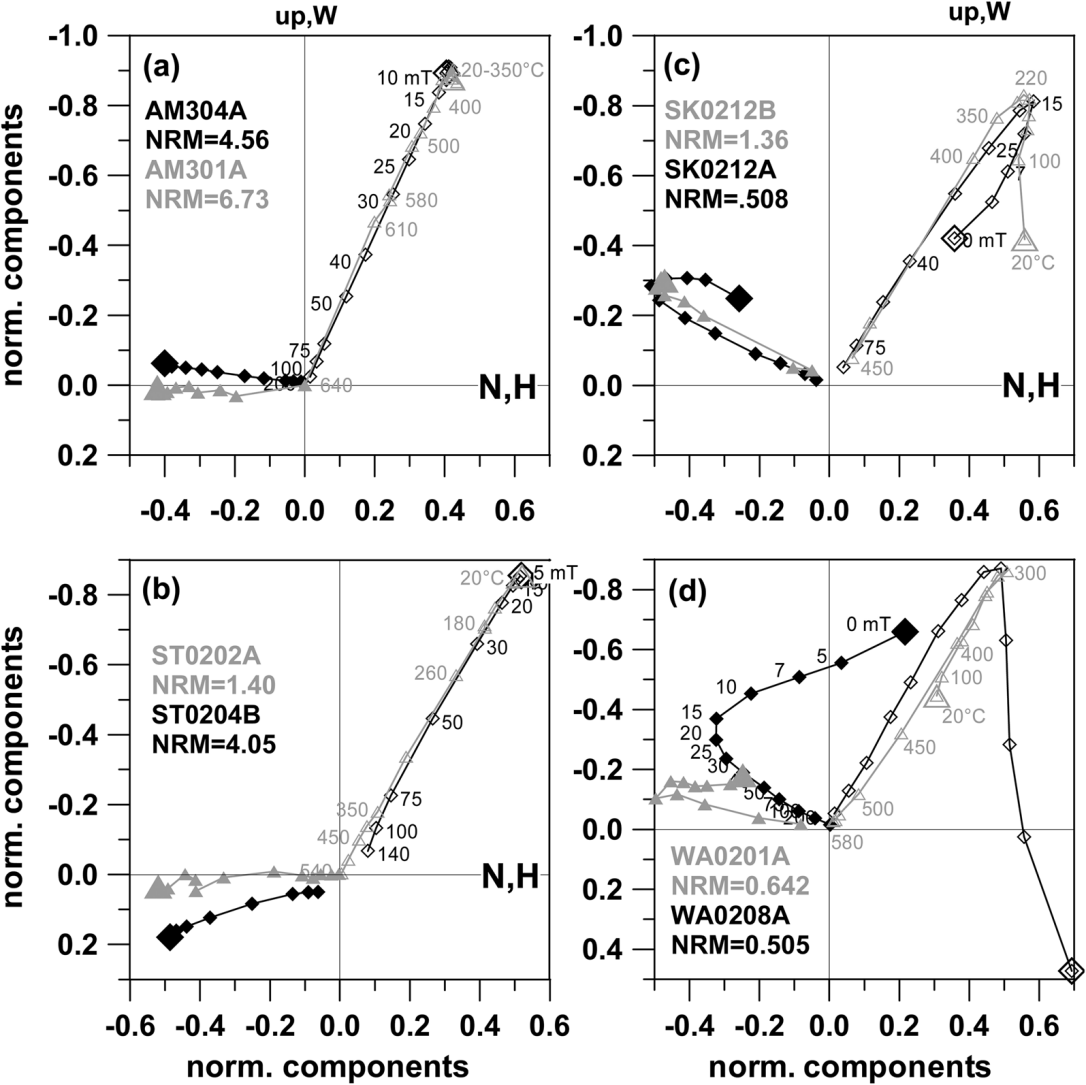
AF (black) and thermal (gray) demagnetization results from a Altenmarkt, b Stein, c Stradner Kogel and d Waltra sites in orthogonal projections. NRM is indicated by a larger symbol. North component is plotted versus East component (closed symbol) and vertical component versus horizontal component (solid symbol). Numbers give demagnetization steps in mT or °C. Specimen names are indicated and NRM intensity is given in A/m

Figure 4a shows an almost ideal demagnetization behavior for site Altenmarkt without secondary components and a very good agreement between AF and thermal data. Site Stein (Fig. 4b) features only weak, easily removable secondary components and the ChRM directions are very well clustered (Additional file 6: Fig. S2). Sites Stradner Kogel (Fig. 4c) and Waltra (Fig. 4d) feature mostly clustered NRM directions and nearly antiparallel overprints, most likely originating from of the present geomagnetic field. Specimens with NRM directions far from the ChRM cluster have strong overprints which were removed with 5 to 30 mT AF demagnetization (e.g., Fig. 4c, d).

**Fig. 5:**
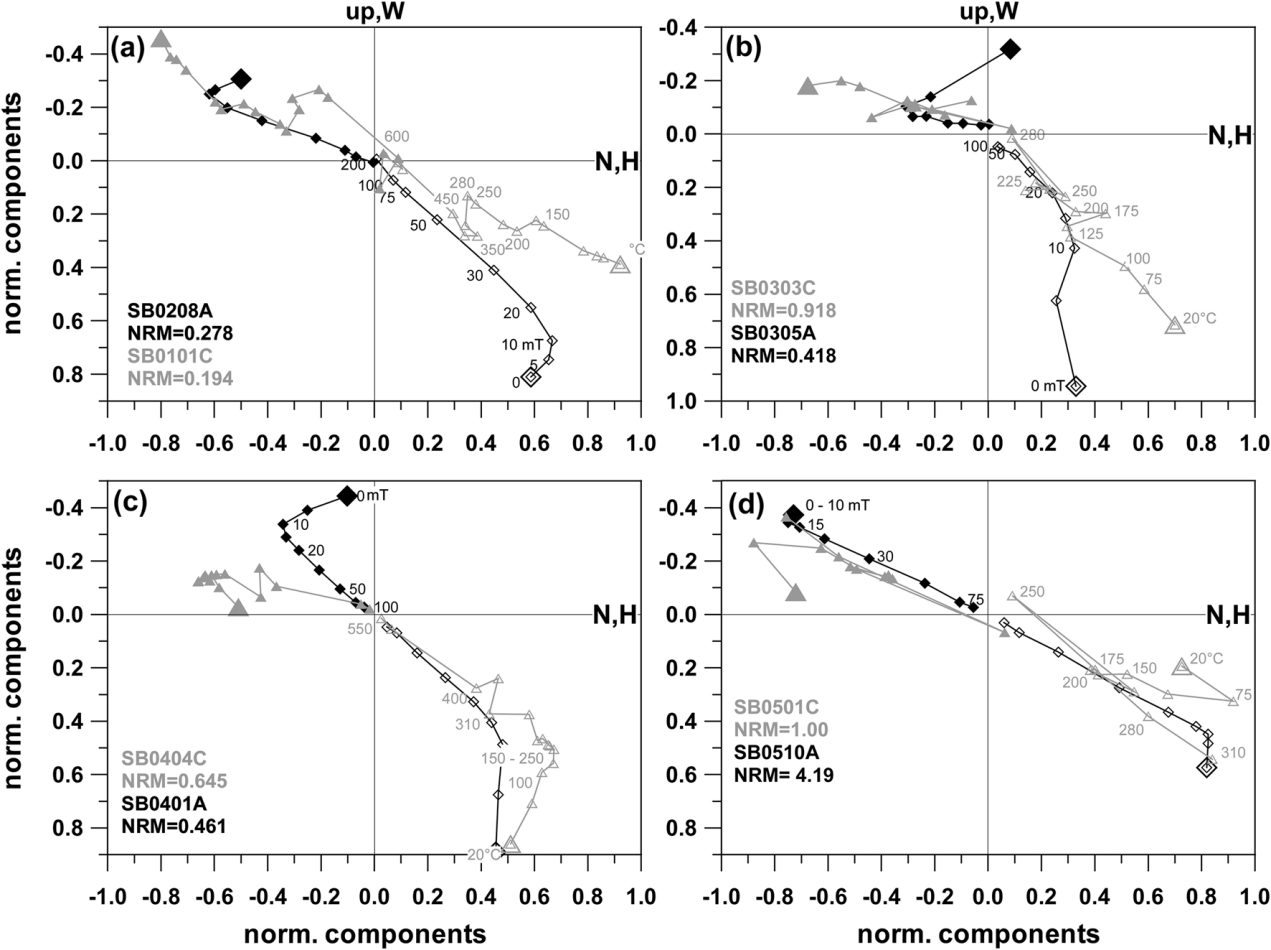
Demagnetizations curves from the Steinberg site. Same representation as in Fig. 4

Five sites were sampled at Steinberg volcano within a big quarry and NRM directions are very scattered with normal inclinations (Additional file 6: Fig. S2, SB01 to SB05). With the exception of two specimens, 5–30 mT or 75–200 °C were needed to remove secondary components (Fig. 5). Comparisons of these treatments with the median destructive field (MDF) and median destructive temperature (MDT) reveal that a large part of the magnetization was affected by secondary components (Additional file 7: Fig. S3, appendix: left). Scattered directions were often obtained during thermal demagnetization (Fig. 5b–d) along with chemical alteration, as suggested by a magnetization increase around 300 °C (e.g., Fig. 5a). Some specimens (13%) were completely unstable and did not yield any ChRM. Valid ChRM directions are similar for all sites, but show a considerable dispersion (Table 1, Additional file 6: Fig. S2, SB01 to SB05).

**Fig. 6:**
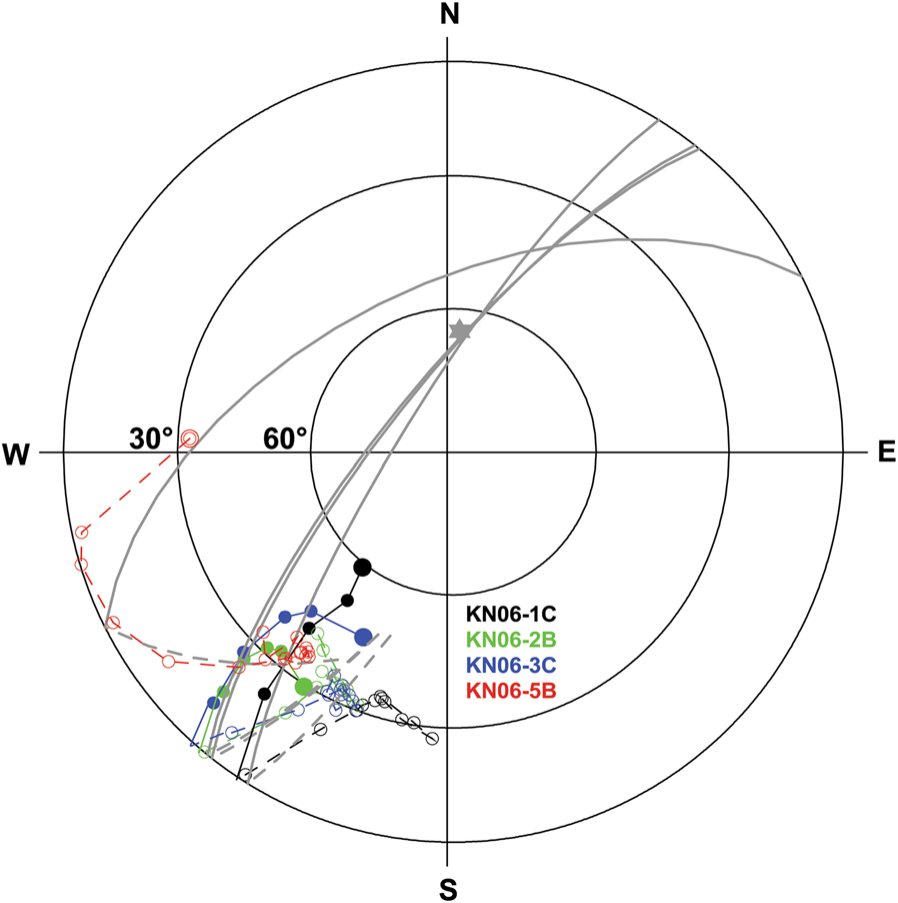
Equal area projection of AF demagnetization data obtained from Klöch. Open symbols represent negative inclinations, closed symbols positive. The NRM is indicated by a larger symbol. Great circles fitted to the secondary component are shown in gray. The gray star gives the present field direction

**Fig. 7:**
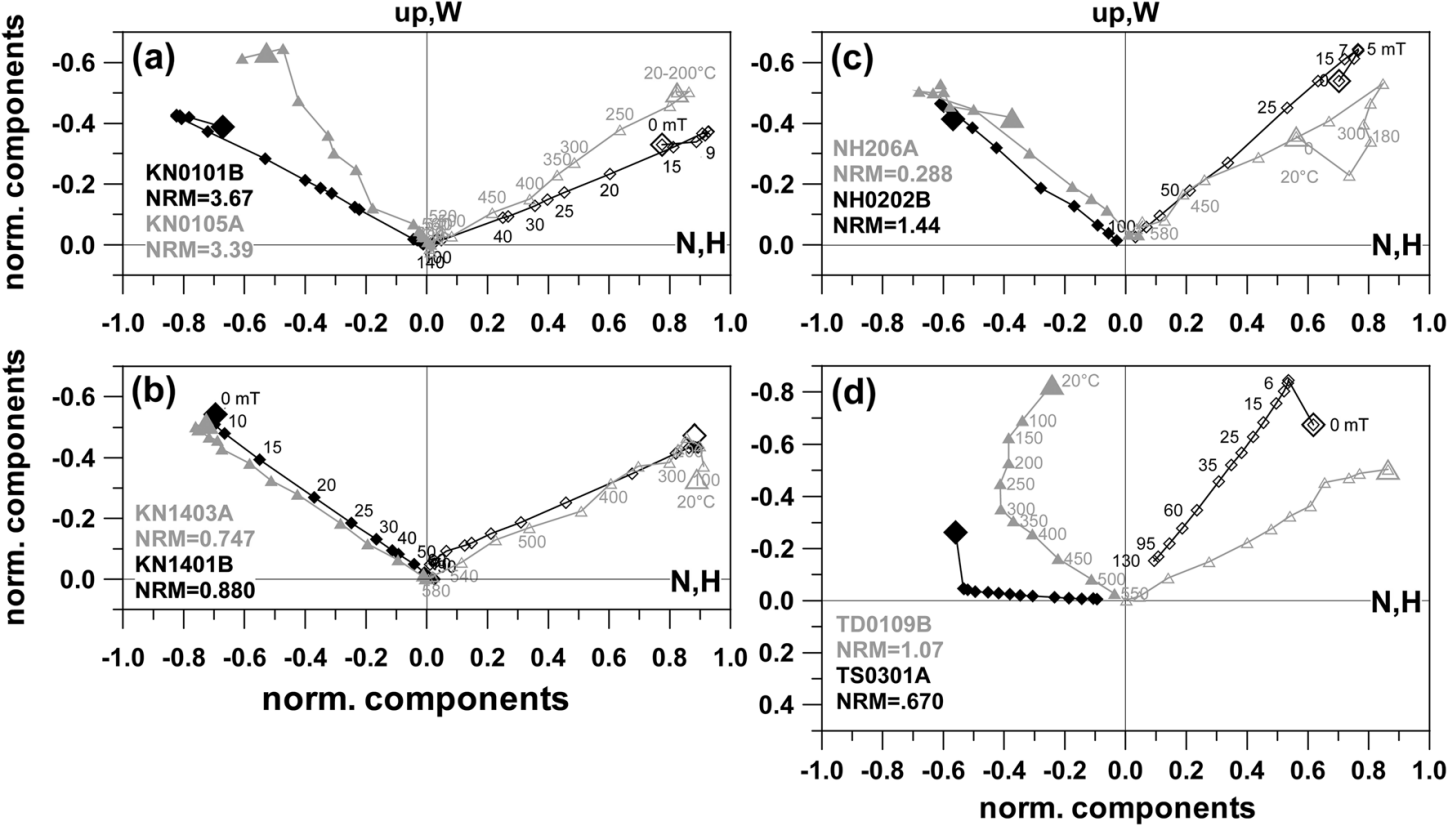
Demagnetizations plots for **a** Klöch quarry, **b** Zaraberg, **c** Neuhaus and **d** Tieschen sites. Same representation as in Fig. 4

Directions of the Klöch quarry and at Zaraberg sites are often affected by some scatter, with well-clustered ChRM directions being observed only in 2 cases (KN08, and KN15). Strong overprints were encountered in most of the demagnetization experiments. AF demagnetization with 5 to 30 mT was sufficient to remove these secondary components, while elevated temperatures up to 380 °C were needed thermal demagnetization. Here MDT and MDF values (Additional file 7: Fig. S3, appendix: right) are generally higher than for Steinberg and some of the Klöch-sites have a good thermal stability with MDT values above 400 °C. The origin of the secondary components was tested by great circle analysis with examples plotted in Fig. 6. They show that the demagnetization path leads to the present field direction. Accordingly, these overprints are viscous magnetizations caused by the present geomagnetic field. This was often but not always the case. Figure 7a, b shows demagnetization examples from Klöch for those sites which had only weak secondary components. The overprints are approximately antiparallel with an increase of intensity at the beginning of demagnetization, followed by straight lines with consistent directions. All sites from Klöch and Zaraberg have unusual ChRM directions with relatively low negative inclinations and declinations between 210° to 230° (Additional file 6: Fig. S2). The samples from the synvolcanic lake sediment layer (Table 1, KN10) showed well-defined clustered directions after removal of the viscous overprint, which are similar to those of the volcanic sites (Additional file 6: Fig. S2). Similar directions were also observed for the lavas from Neuhaus and for three dikes in Königsberg north of the Klöch volcano (Additional file 6: Fig. S2, NH02 and TD01, TD02 TS03/TD04). The examples show moderate secondary overprints (Fig. 7c, d) and well-defined ChRM directions. Considerable directional scatter among samples is observed for all sites (Klöch, Zaraberg, Königsberg and Neuhaus) with these unusual directions (see Additional file 6: Fig. S2).

In summary, 28 well-defined ChRM directions have been obtained from 164 AF and 111 thermal demagnetization experiments. Moreover, paleointensity experiments (see below) provided further 49 ChRM directions from the oriented specimens and mini-cores. The site mean directions were calculated hierarchically, averaging specimens of independently oriented samples first, and then sites using Fisher (1953) statistics. The statistical parameters and the mean ChRM directions are given in Table 1 and they are shown together with their confidence circles α_95_, NRM directions of specimens and ChRM directions of samples in Additional file 6: Fig. S2. For all sites the scatter of NRM directions was considerable reduced by the demagnetizations and well-defined mean ChRM directions are based on 5 (in one case only 3) to 18 independently oriented samples. Sites with unusual directions tend to have larger α_95_ circles.

### Paleointensities

Evaluation of the paleointensity experiments started with determination of the ChRM direction, which failed for 11 specimens (Additional file 1: Table S1). A trend towards the laboratory field direction was also often observed. The ThellierTool4.22 software (Leonhardt et al. 2004) with default parameters for Classes A and B was used for preliminary evaluation and the results were checked carefully and sometimes adjusted, for instance by removing initial heating steps when they were obviously affected by an overprint. A few specimens, slightly exceeding class B criteria but providing a good straight line fit, were also accepted (Class C). Another 27 specimens failed to give acceptable results.

For further characterization of our 37 results, additional parameters were calculated, such as the NRM fraction FRAC, the Arai plot curvature $\left|{\overrightarrow{\text{k}}}{^{\prime}}\right|$, and the correlation coefficient ${\text{R}}_{\text{corr}}^{2}$ (Additional file 1: Table S1, for definitions see Paterson et al. 2014, 2015). These parameters were checked with the software of Béguin et al. (2020). Three sets of criteria are used for the final classification (Table 2): the strict (S) criteria were based on the MC-CRIT.B1 set of Paterson et al. (2015). The same parameters are used for the moderate (M) criteria based on the modified TTB set of Paterson et al. (2014). The third set takes the rather weak (W) criteria of Bono et al. (2019). Here only very basic requirements are fulfilled: A minimum of 5 steps is used for calculation of the slope in the Arai plot and linearity of the fit line is achieved by a correlation of *R*^*2*^ ≥ 0.9; the zero field steps must have a trend to the origin with a maximum angular dispersion of < 10° and alteration assessed by PTRM checks must be < 10%.

**Table 2:** Criteria sets were used for classification of the paleointensity results

Criterion	Strict (S)	Moderate (M)	Weak (W)	Range
*n*	** ≥ **5	** ≥ **5	** ≥ **5	5–15
FRAC	** ≥ **0.45	** ≥ **0.30	**–**	0.19–0.97
*β*	** ≤ **0.1	** ≤ **0.1	**–**	0.01– 0.15
*q*	** ≥ **5	** ≥ **3	**–**	1.6–52.7
*|*$\overrightarrow{\text{k}}$*'*|	** ≤ **0.13	** ≤ **0.48	**–**	0.00–1.01
MAD_Anc_	** ≤ **6	** ≤ **10	** ≤ **10	1.1–5.8
α	** ≤ **10	** ≤ **15	**–**	0.6–14.3
DRAT	**–**	**–**	** ≤ **10	1.5–11.0
δCK	** ≤ **7	** ≤ **9	**–**	1.2–6.8
δpal	** ≤ **9	** ≤ **18	**–**	0–40.8
δTR	** ≤ **3.4	** ≤ **20	**–**	1.4–13
Δt*	** ≤ **9.0	** ≤ **50	**–**	0–7.3
${\text{R}}_{\text{corr}}^{2}$	**–**	**–**	** ≥ **0.9	0.915–1.000
Reference	Paterson et al. (2015)	Modified from Paterson et al. (2014)	Bono et al. (2019)	

*n*: number of data points used for the linear fit; FRAC: NRM fraction; *β*: standard deviation of slope; *q*: quality factor (Coe et al. 1978); |$\overrightarrow{\text{k}}$'|: Arai plot curvature; MAD_Anc_: maximum angular deviation of anchored line; *α*: angular difference between anchored and not-anchored solution; DRAT: deviations of pTRM checks; δCK: pTRM checks; δpal: cumulative check difference; δTR: relative intensity difference of tail checks; Δt*: normalized tail of pTRM; ${\text{R}}_{\text{corr}}^{2}$: correlation coefficient of fit line (for definitions see Paterson et al. 2014 and references therein). The last column gives the range of the values obtained for the successful experiments

**Fig. 8:**
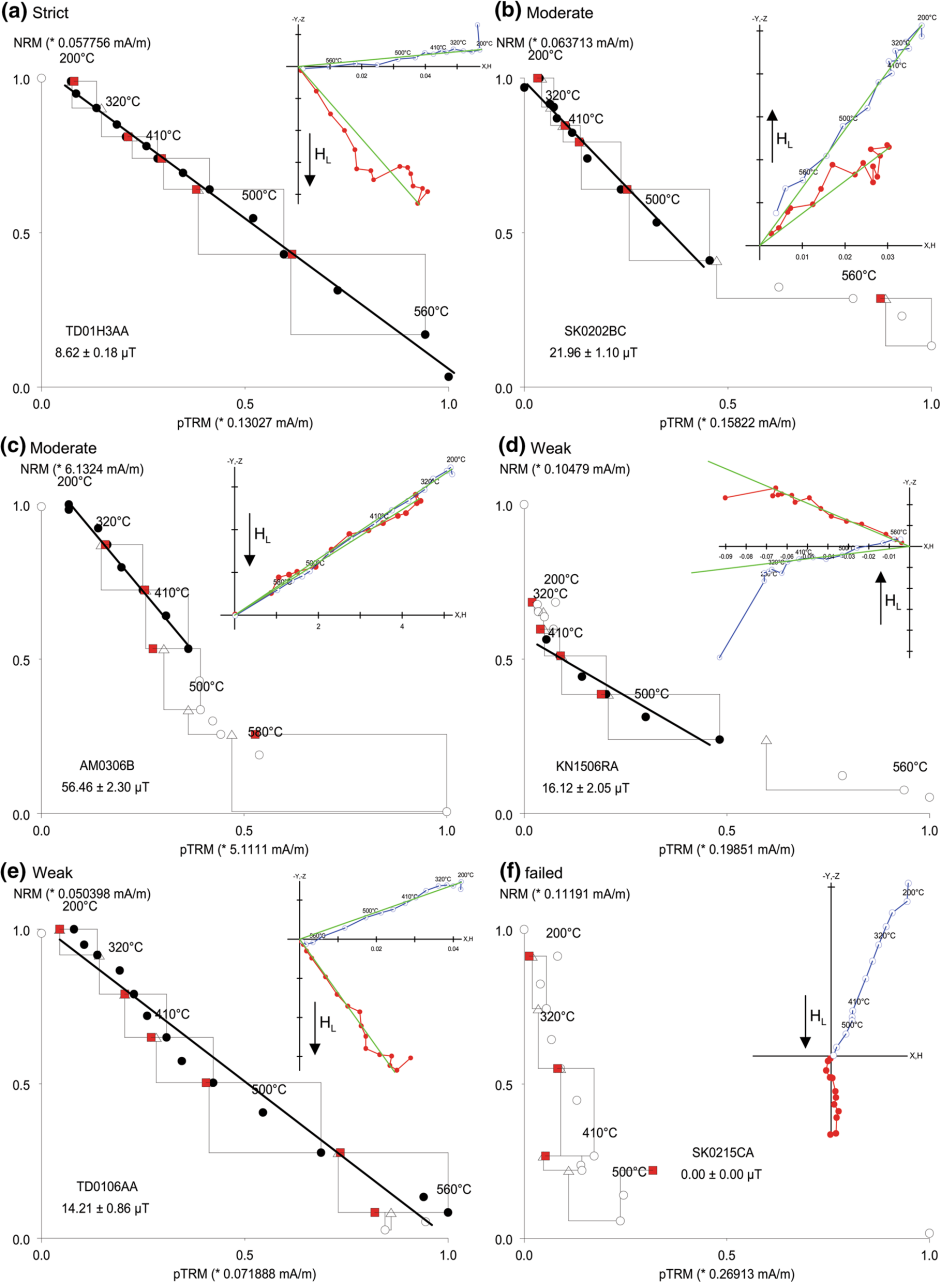
Examples of Thellier–Coe experiments shown in Arai plots with pTRM checks as triangles, and accepted steps as dots. The insets show orthogonal vector component projections in sample coordinates (vertical plane: open, blue symbols; horizontal plane: solid, red symbols, green line: mean direction associated with the chosen temperature interval). Further information can be found in Additional file 3: Table S3 and Additional file 8: Fig. S4

Representative examples for each set of criteria and one failed experiment are given in Fig. 8. Only 3 specimens fulfill the strict criteria (Fig. 8a), with very good linearity of the Arai plot and in the orthogonal vector plot of demagnetization, positive pTRM, additivity and tail checks and a very small error of the obtained paleointensity (Additional file 1: Table S1). Moderate criteria were fulfilled by 24 specimens. About half of them have also a good Arai plot linearity, but they do fail one to three of the S criteria, which are often δpal or δTR and sometimes $\left|{\overrightarrow{\text{k}}}{^{\prime}}\right|$ or FRAC. The other specimens fulfilling M criteria show concave Arai curves with an increase of TRM capacity and a trend towards the laboratory field direction, and in one case a decrease of TRM capacity followed by an increase (Fig. 8b, c). In these cases, slightly curved Arai plots were accepted over ranges where the alteration was still very low, as indicated by matching pTRM and additivity checks. Accordingly, the main parameters responsible for S criteria failures were $\left|{\overrightarrow{\text{k}}}{^{\prime}}\right|$ and in a few cases FRAC, δTR, δpal or q. Ten specimens that met only the weak criteria are not particularly different in their behavior (Fig. 8d, e), but they do fail for $\left|{\overrightarrow{\text{k}}}{^{\prime}}\right|$ or FRAC and often they bear a considerable secondary component (Additional file 8: Fig. S4d). Some specimens failed because of a strong TRM capacity decrease above 400 °C, which was followed by an increase due to the formation of new minerals (Fig. 8f).

### ^*39*^*Ar/*^*40*^*Ar-dating*

Ar release spectra from incremental heating were obtained for 10 samples from 2 volcanic localities. Spectrum analysis criteria used to determine the sample ages include at least 3 overlapping consecutive steps (95% confidence), accounting for > 50% cumulative ^39^Ar release. An inverse isochron age was then calculated from these release steps and we use those results as sample ages. We chose this strategy since the isochron method does not contain any assumption regarding the trapped atmospheric contaminant.

The gas release spectra with inverse isochrons are displayed in Table 3 and Additional file 9: Fig. S5. The release spectra of all samples from Klöch contain a plateau. Inverse isochron results yield an inverse weighted mean age of 2.51 ± 0.27 Ma (MSWD: 0.46, P: 0.71). Only 3 samples from Steinberg met the aforementioned age criteria (SB0202A, SB0403A and SB404A) with an inverse weighted mean age of 2.46 ± 0.12 (MSWD: 0.72, P: 0.49).

**Table 3:** ^39^Ar/^40^Ar-dating results of the Styrian basalts

Location	Sample	Material	Steps (n)	Spectrum					Inverse Isochron			
				%^39^Ar	Age ± 1.96 s (Ma)	MSWD (P)	TGA ± 1.96 s	K/Ca ± 1.96 s	Age ± 1.96 s (Ma)	MSWD (P)	Trapped ^40^Ar/^36^Ar	Spread (%)
Klöch	KN01-2B	Groundmass	5–15 (11)	70.06	2.92 ± 0.27	0.94 (0.50)	3.61 ± 0.32	0.41 ± 0.02	**2.89 ± 0.92**	1.04 (0.40)	299.01 ± 15.90	7.5
	KN06-3C	Groundmass	3–10 (8)	67.74	3.11 ± 0.88	0.36 (0.93)	3.77 ± 0.99	0.80 ± 0.09	**2.14 ± 1.67**	0.20 (0.98)	307.33 ± 15.33	11.5
	KN07-5D	Groundmass	2–9 (8)	93.79	2.65 ± 0.23	0.52 (0.82)	3.11 ± 0.35	0.03 ± 0.0005	**2.20 ± 0.83**	0.43 (0.86)	316.77 ± 35.90	18.5
	KN08-5B	Groundmass	1–18 (18)	100	2.70 ± 0.24	0.57 (0.92)	2.82 ± 0.29	0.15 ± 0.01	**2.52 ± 0.31**	0.41 (0.98)	303.18 ± 5.18	35.1
Weighed mean									**2.51 ± 0.27**	0.46 (0.71)		
Steinberg	SB0202A	Groundmass	1–13 (13)	83.07	2.39 ± 0.04	0.56 (0.88)	2.12 ± 0.18	1.78 ± 0.02	**2.54 ± 0.25**	0.49 (0.91)	289.12 ± 15.49	21.7
	SB0401A	Groundmass	2–4 (3)	44.7	2.39 ± 0.05	1.23 (0.29)	2.70 ± 0.17	2.27 ± 0.04	2.36 ± 0.06	1.00 (0.00)	299.83 ± 1.93	25.9
	SB0402A	Groundmass	1–20 (20)	100	2.88 ± 0.38	32.20 (0.00)	3.11 ± 0.11	0.06 ± 0.00	2.76 ± 0.45	40.89 (0.00)	300.44 ± 11.95	44.7
	SB0403A	Groundmass	2–19 (18)	78.18	2.49 ± 0.07	0.44 (0.98)	3.05 ± 0.68	0.05 ± 0.00	**2.45 ± 0.14**	0.44 (0.97)	305.29 ± 22.93	61.6
	SB0404A	Groundmass	1–13 (13)	100	2.16 ± 0.79	0.14 (1.00)	2.61 ± 1.29	0.03 ± 0.00	**2.03 ± 0.83**	0.11 (1.00)	305.13 ± 18.32	50
	SB0405A	Groundmass	1–18 (18)	100	2.39 ± 0.38	13.34 (0.00)	2.33 ± 0.12	0.10 ± 0.00	2.25 ± 0.46	19.12 (0.00)	299.57 ± 7.69	35.4
Weighed mean									**2.46 ± 0.12**	0.72 (0.49)		

The gas release spectra with plateau age and inverse isochron ages are given. Only reliable inverse isochron ages (bold) were accepted for calculations of the weighted mean ages (bold). Location and sample names correspond to Table 1

## Discussion

### Mean paleomagnetic directions and virtual geomagnetic poles

Most specimens showed weak-to-strong secondary overprints. In almost all cases, an increase of the intensity at the beginning of the demagnetization experiments suggests that the secondary component has an approximately antiparallel direction. Because all sites have ChRM directions nearly opposite to the present-day field, secondary components are most likely viscous overprints. This is supported by the fact that, for many specimens, great circles fitted to the secondary component run through the present field direction. The magnetization increase with respect to the NRM was considerable, often exceeded 30% of the initial NRM intensity. Accordingly, specimens with a pure primary TRM, which is a prerequisite for paleointensity determination, were rare.

**Fig. 9:**
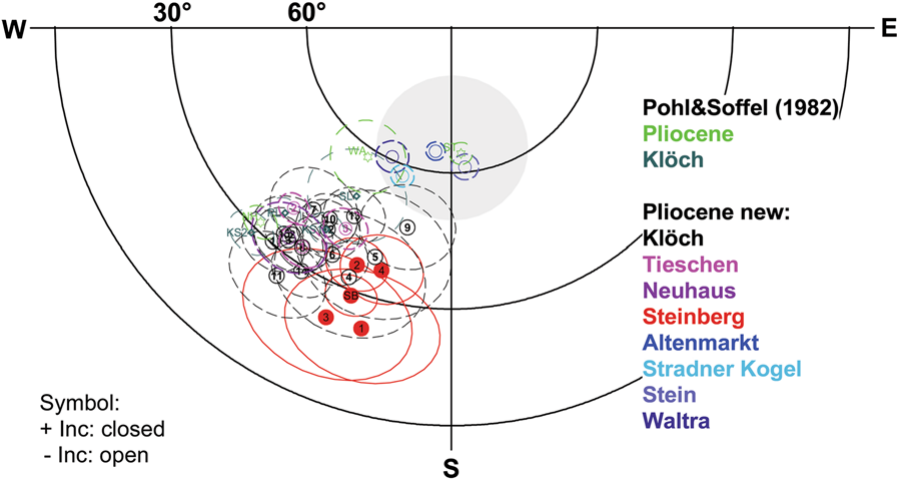
Mean characteristic remanent magnetzations and α_95_ of the Styrian volcanic rocks. The gray area shows the expected of secular variation. Data from Pohl and Soffel (1982) are shown for comparison

Secondary components were mostly removed with ≤ 30 mT AF demagnetization, while 300 °C or even more were required during thermal demagnetization. In most cases well-defined mean ChRM directions were obtained (Table 1) with values of the precision parameter *k* (Fisher 1953) ranging from 11 to 1276, and α_95_ values between 2° and 15°. Sites having strong secondary overprints indicated by large dispersions of the NRM directions (cf. Table 1 and Additional file 6: Fig. S2, e. g. Klöch quarry: KN11 or Steinberg: SB01 to SB05) often yield mean ChRM directions with relatively low k values. The mean ChRM directions of only 4 sites are reversed and within the range of secular variation (Fig. 9). The remaining sites show anomalous directions confined to the from 190 to 240° declination sector. Sixteen sites have low negative inclinations and 5 sites have low positive inclinations. The confidence circles are generally larger for sites with intermediate directions and the large dispersion of NRM directions indicate outweighing of strong secondary components. Nevertheless, magnetic cleaning was successful and provided well-defined reliable mean ChRM directions.

**Fig. 10:**
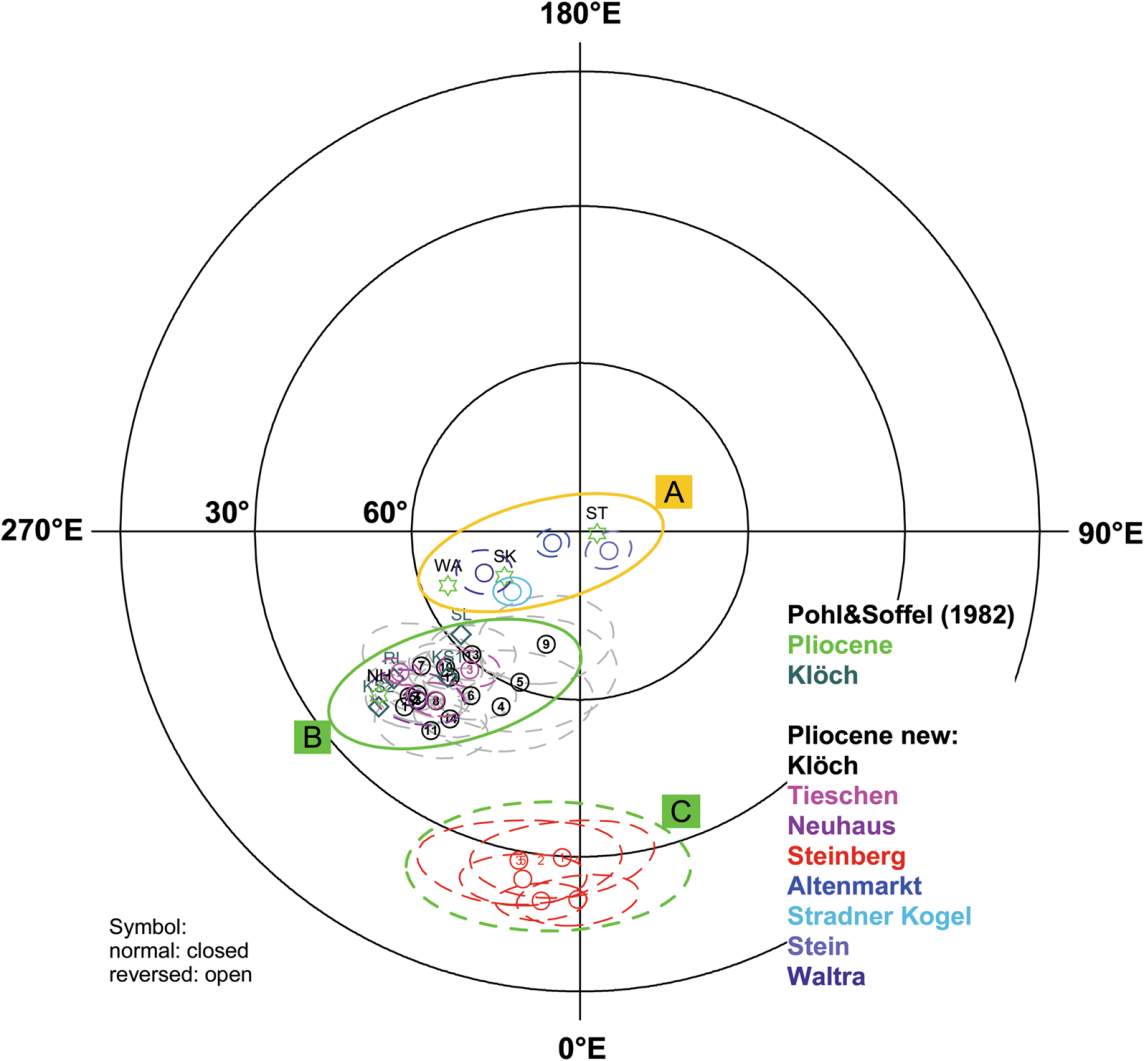
Mean virtual geomagnetic pole (VGP) positions obtained in this study (circle, with error ellipses) in comparison with data of Pohl and Soffel (1982, green stars). Three clusters were identified (see text for details)

**Fig. 11:**
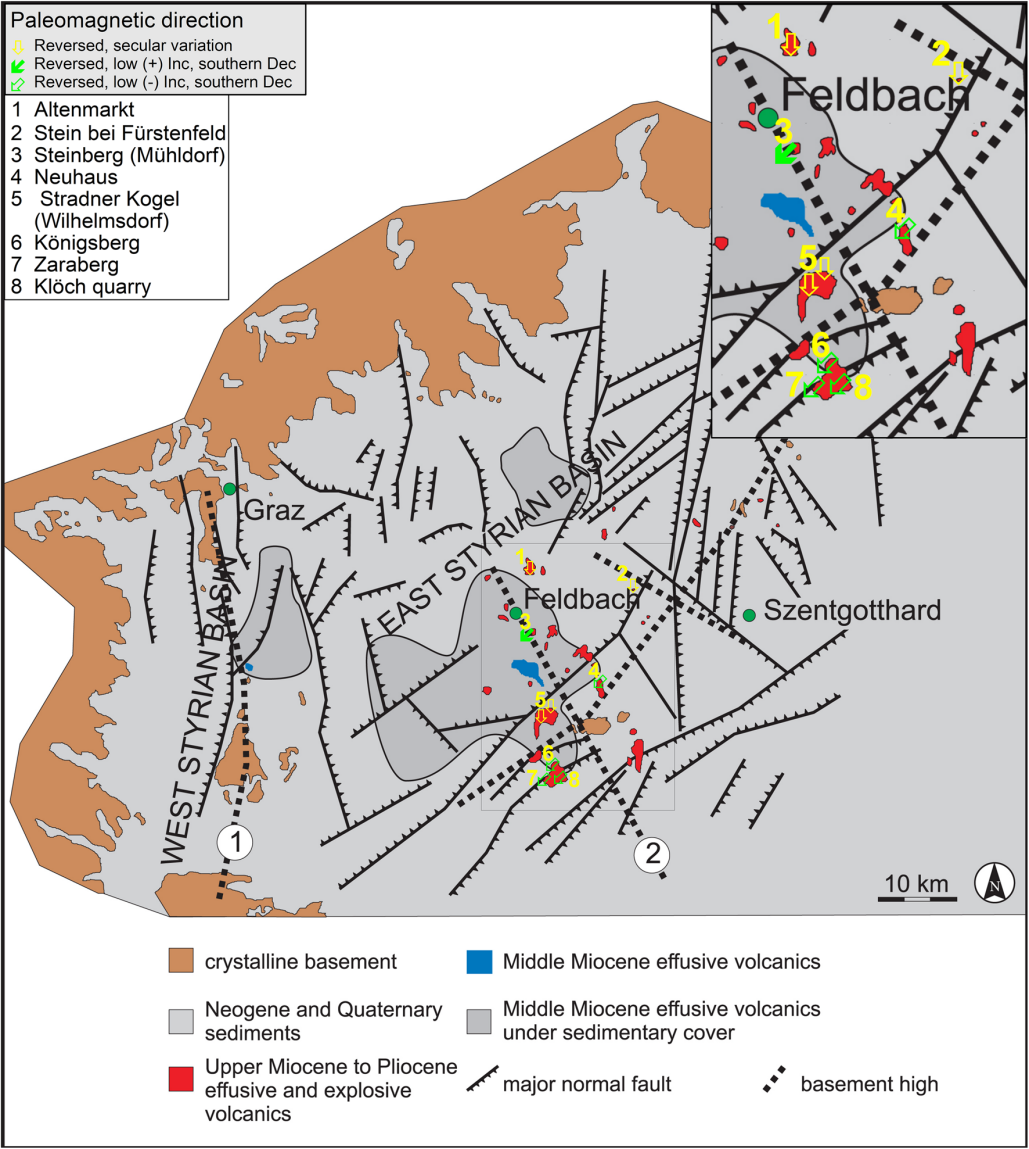
Map of the Styrian basin showing main geological units, and tectonic and volcanic features ( modified from Bojar et al. 2013). Paleomagnetic sites and results are included

Calculation of the reversal angle δ (Prévot et al. 1985) and the virtual geomagnetic poles (VGP, Table 1 and Fig. 10) shows that all sites have reversed polarities and only 4 sites cluster close to the geographic pole having high VGP latitudes (< − 70° see Table 1, cluster A in Fig. 10) and a reversal angle δ < 25°. Furthermore, VGPs are mainly confined in a narrow longitudinal sector from 290 to 360°E. Two more clusters with low VGP latitudes can be identified, one (cluster B) at about – 45° and another (cluster C) below − 30°. Other sites from Klöch and Zaraberg investigated by Pohl and Soffel (1982) fall also in this cluster and the poles of Pohl and Soffel (1982) are well reproduced (Table 1; Fig. 10). While for cluster C δ angles exceed 80° for cluster B they range from 18.9° to 46.2°. This VGP distribution obviously does not represent secular variation. Accordingly, the possibility of tectonic movements must be taken into consideration. In this case, the recorded anomalous directions would require a ~ 30° rotation of the Klöch–Königsberg and Neuhaus volcanoes and an even larger rotation for Steinberg. The lavas of two volcanoes are well exposed in large quarries and there is no geological evidence for local displacements or regional movements affecting all 5 localities with unusual directions. Sites with directions in the range of SV and those with anomalous directions are distributed all over the volcanic field and no correlation with tectonic features is found (Fig. 11). For instance, Klöch and Königsberg are separated by a normal fault, but the paleomagnetic directions are very similar. Furthermore, the synvolcanic lake sediment layer (Table 1, KN10) yielded a ChRM direction within the cluster of the Klöch–Königsberg lavas. The detritus came very likely from volcanic ejection of the Königsberg, which implies also a stratigraphic constraint between the Klöch and Königsberg lavas. The sediment is layered nearly horizontally, displays the visible underlying morphology and does not give evidence for local tectonic movements. Furthermore, the similar directions of Klöch–Königsberg complex and Neuhaus volcano would require the same local tectonic movement to explain their unusual but very similar directions, which is rather unlikely. Finally, the Miocene Gleichenberg volcano (in blue in Fig. 11) recorded normal polarity directions in the range of secular variation (Pohl and Soffel 1982). Accordingly, a large-scale tectonic rotation after the Miocene can be excluded for the area.

Therefore, the only remaining conclusion is that the Styrian volcanoes recorded intermediate field directions. Following Prévot et al. (1985), a reversal angle δ > 25° can be attributed to a transitional field direction. Accordingly, the sites of cluster C are clearly transitional. However, three sites (KN09, KN13, SL) in cluster B have δ < 25° (Table 1). Due to their close temporal proximity to the other sites of cluster B recording of a transitional field configuration seems to be plausible. This hypothesis was further tested by investigation of the paleointensity.

### Paleointensity

Paleointensity determination was difficult, due to secondary components and chemical alteration during heating. Thellier experiments were performed for 15 sites and the failure rate was about 50%. Only 3 specimens fulfill the strict MC-CRIT.B1 criteria set of Paterson et al. (2015), while for the moderate criteria set, the NRM fraction FRAC was lowered to 0.3, considerably less than recommended by Paterson et al. (2014, 2015: 0.45). FRAC (see Shaar and Tauxe 2013) was introduced instead of the fraction factor *f* (Coe et al. 1978) because *f* may be overestimated in case of strongly concave Arai plots. This behavior is caused by the presence of a large amounts of MD particles, but also by strong viscous overprints. Viscous overprints have been often observed during our paleointensity experiments, for instance for the specimen shown in Fig. 8d, which lost about one-third of the NRM during the first heating step in combination with AF demagnetization. This loss was accompanied by a strong directional change. *κ(T)* (Additional file 5: Fig. S1) gives no indication for the presence of a mineral with low Curie temperature. While the secondary component is still present until 350 °C, very little TRM is gained in the Thellier experiment. The viscous component was relatively large compared to the paleo-TRM because this TRM was acquired in a much weaker field than the present-day value. The fraction factor *f* is > 0.5 for this example (Additional file 1: Table S1) and, although somewhat overestimated, *f* appears to be more appropriated than FRAC in such cases. The hysteresis parameters of this specimen point also to a large MD fraction, which explains the strong curvature $\left|{\overrightarrow{\text{k}}}{^{\prime}}\right|$ in the Arai plot. Mineral alteration starts at ~ 500 °C (Additional file 5: Fig. S1), as shown by the last pTRM check. Accordingly, the selected data points give a reasonable paleointensity estimate and this is also the case for the example in Fig. 8 e. It is obvious that most of our samples do not have magnetic properties which are suited to provide very precise paleointensities. Instead, the aim of the study is rather to find support for the intermediate character of the directions by intensity measurements and thus to have more indications for a transitional field configuration. Hence, many of the results which we take into consideration allow only for a rough assessment of paleointensity and do not fulfill the various published and widely accepted more strict criteria (e.g., Kissel and Laj 2004,2015; Biggin et al. 2007; Paterson et al. 2014; Cromwell et al. 2015; Tauxe et al. 2016). Although such reliability criteria sets are undoubtedly useful for statistical investigation for long-term trends of the field, a too rigorous application rejects many experiments which could yield slightly uncertain, but important information about the paleofield configuration.

Finally, paleointensity results from 10 sites are taken into consideration and 9 mean paleointensity values have been calculated (Table 4), in one case by combining single values for 2 sites from Steinberg volcano. These mean values are robust and remain within their error margins when a stricter selection (only strict and moderate criteria accepted) is made (Additional file 1: Table S1).

**Table 4:** Paleointensity results of the Styrian lavas

Location	Latitude (°N)	Longitude (°E)	Name	D (°)	I (°)	α_95_ (°)	Plat (°N)	PLong (°E)	PI (µT)	sPI (µT)	VDM (10^22^ Am^2^)	sVDM (10^22^ Am^2^)
Altenmarkt	47.005	15.912	AM03	187.6	− 64.3	1.9	− 84.7	293.7	57.4	3.4	9.3	1.1
Stradner Kogel	46.841	15.926	SK02	198.5	− 57.6	2.3	− 73.9	311.6	22.0		3.9	
Waltra	46.849	15.952	WA02	204.8	− 60.6	3.3	− 71.5	293.4	17.4	0.5	3.0	0.2
Klöch 12	46.767	15.968	KN12	211.6	− 40.1	6.2	− 55.2	318.3	12.0	2.0	2.6	0.8
Klöch 14	46.764	15.946	KN14	211.8	− 28.8	5.7	− 48.9	325.2	8.6	0.7	2.0	0.3
Klöch 15	46.764	15.946	KN15	219.2	− 33.5	3.8	− 47.1	314.1	12.9	4.2	2.9	1.9
Tieschen 01	46.783	15.954	TD01	214.4	− 33.7	4.5	− 50.1	319.4	11.2	2.5	2.6	1.1
Tieschen 03	46.787	15.956	TS03/TD04	207.9	− 42.2	4.3	− 58.4	321.4	10.5	3.9	2.2	1.6
Steinberg SB	46.935	15.917	SB01-05	200.7	28.8	4.7	− 25.0	353.8	4.9	2.6	1.1	1.2

*D*: declination of the ChRM; *I*: inclination of the ChRM; α_95_: 95% confidence circle radius of the Fisher (1953) statistics; Plat: latitude of the VGP position; Plon: longitude of the VGP position; PI: mean paleointensity; sPI: standard deviation of paleointensity; VDM: virtual dipole moment from PI; sVDM: standard deviation of VDM

**Fig. 12:**
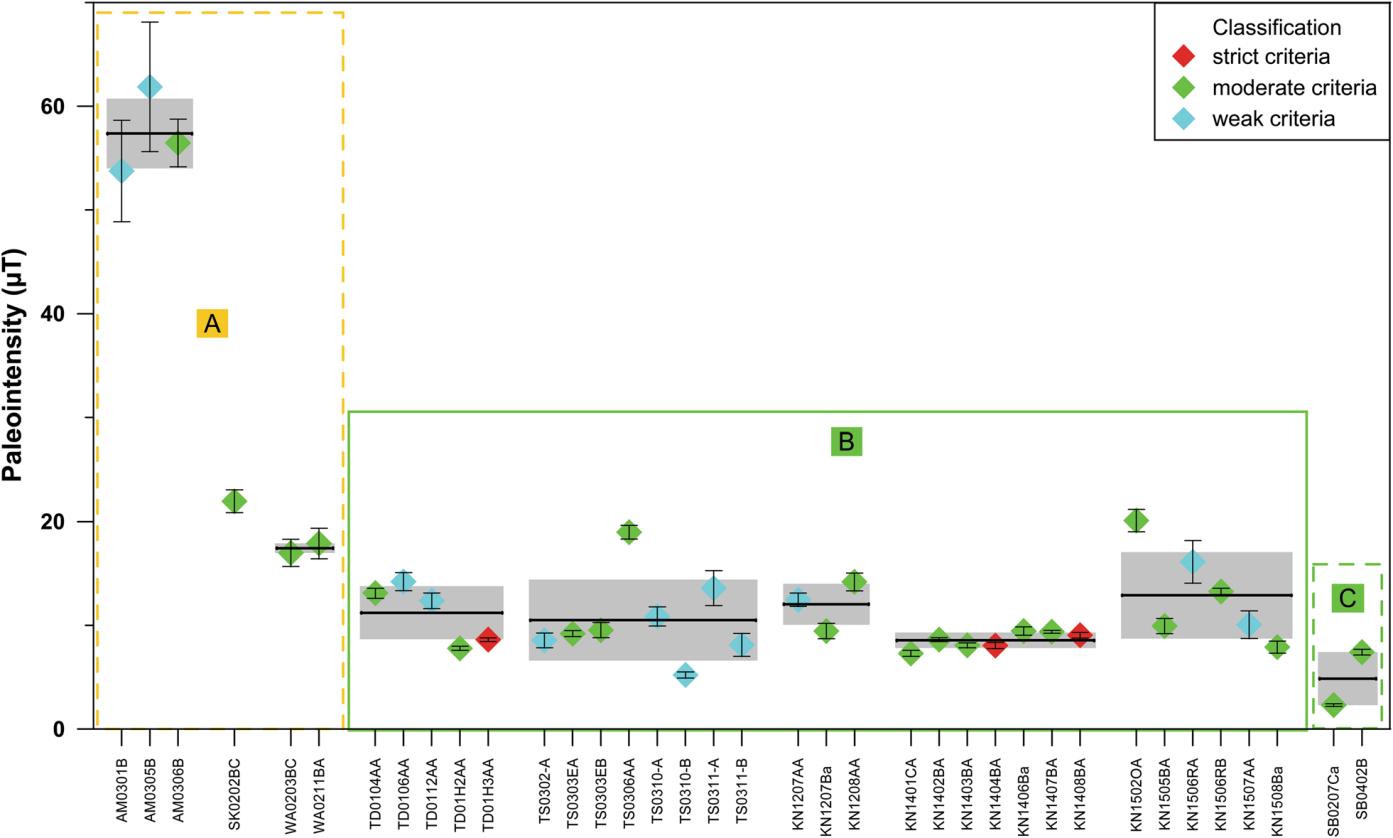
Summary of paleointensity results. Individual values and site means are plotted with error bars/bands versus specimen names for the 3 VGP clusters (see Fig. 10)

Figure 12 shows a summary of all accepted paleointensity results together with the site means. A large variation of paleointensities is seen between the sites, while the within-site dispersion is considerably smaller.

Data in Fig. 12 are grouped according to the above described VGP clusters. Class M paleointensity results are present in all groups and the class W results do not show larger deviations. Paleointensities of sites with low VGP latitudes lie in the range from 8 to 13 µT and only 5 µT has been determined for the lowest VGP latitudes. These values are relatively low compared with the field intensity which was on average about 35 ± 8 µT for the past 5 Ma (Muxworthy 2017). While one site with high VGP latitude has a relatively large intensity of 57 µT, the values from the Stradner Kogel sites are only around 20 µT and not much higher than those of the transitional sites. The Stradner Kogel VGPs lie relatively close to cluster B and the reversal angles are above 10°. This could suggest that the Stradner Kogel sites were formed shortly before or after the sites of cluster B. Nevertheless, a rough correlation is seen between VGP latitude and paleointensity or virtual geomagnetic dipole moment (VDM, see Table 4). This strongly supports the hypothesis that the low-latitude VGPs were recorded during a transitional or excursional field configuration.

### Ages and correlation with geomagnetic instability time scale

**Fig. 13:**
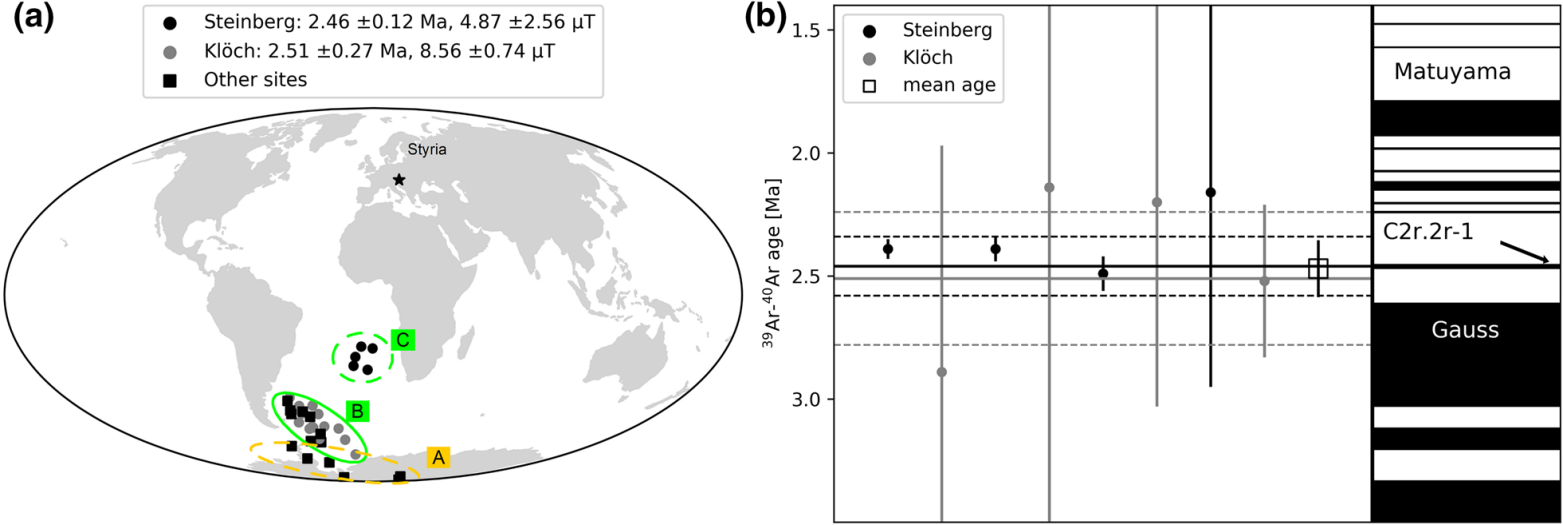
**a** Clusters of virtual geomagnetic poles from Styria. **b** ^39^Ar/^40^Ar-ages with 2 sigma errors from individual samples are shown together with their weighted mean and error band in comparison with the geomagnetic instability time scale (Singer 2014; Ogg et al. 2016)

K/Ar data obtained from lavas or associated tuffs of our sites give an age interval comprised between 2 and 4 Ma for the Pliocene effusive volcanoes in the Styrian basin (Bojar et al. 2013 and references therein, Table 1). New ^39^Ar/^40^Ar-dating performed on samples from 2 localities of our collection was successful for the Klöch and Steinberg volcanoes, which carry transitional directions. They gave weighted locality mean ages of 2.51 ± 0.27 Ma (Klöch) and 2.46 ± 0.12 Ma (Steinberg), respectively. The ^39^Ar/^40^Ar ages (Table 3) agree within their error margins and the age average of 2.47 ± 0.11 Ma allows for correlation with the GPTS (Fig. 13). The transitional lavas are certainly younger than the Gauss–Matuyama reversal and the most likely correspondence is the cryptochron C2r.2r-1 (2.420–2.441 Ma, Cande and Kent 1995). According to Singer (2014; Laj and Channell 2007 and references therein), this is the lowermost geomagnetic excursion in the Matuyama chron and is recorded in marine sediments as well as in terrestrial lava flows on Ouahu, Hawaii (Hawala: ~ 2.51 Ma, Herrero-Bervera et al. 2007; Wheeler Air Force core: ~ 2.46 Ma, Guillou et al. 2018) and on Santiago Island, Cape Verde (2.411 Ma, Knudsen et al. 2009). Guillou et al. (2018) propose 2.46 ± 0.13 Ma as age of cryptochron C2r.2r-1. Channell et al. (2020) emphasize that excursions have synchronous global manifestation in many cases. Accordingly, our transitional directions provide further evidence of cryptochron C2r.2r-1.

### Distribution of VGPs

The VGP positions of our group B lie in the South Atlantic, east of the Falklands, while those of Steinberg (group C) are found east of St. Helena (Fig. 13). Hoffman (2000) identified the South Atlantic and Central Asia as regions for preferred VGP positions of the Matuyama–Brunhes reversal for North Atlantic marine sediments. While different recurrent pole positions west of Australia were found for Tahiti (Hoffman and Singer 2004) and further evidence for this preferred region is presented in a review (Hoffmann et al. 2019) of Cenozoic paleomagnetic transitional field records obtained from lavas in the Southern Hemisphere (e.g., south Indian Ocean, eastern Australia and New Zealand), a subordinate cluster of VGPs in the South Atlantic is present in these studies as well. The situation is different when records from the northern hemisphere are considered (Hoffmann and Singer 2004). Clusters of VGPs in the South Atlantic are found for several transitional records spanning Miocene to Pleistocene ages. They were obtained in the Mediterranean region (Tric et al. 1991; Valet and Laj 1984), the Atlantic Ocean (Riisager et al. 2003, Leonhardt and Soffel 2002) or in California (Glenn et al. 1999). Here the Gauss–Matuyama reversal was recorded. Accordingly, VGP positions associated with transitional field states have been detected in the South Atlantic. Aside from that, the most prominent magnetic anomaly of the present geomagnetic field is situated in South Atlantic and Engbers et al. (2020) provide evidence that anomalous long-term geomagnetic field behavior of this region was already present ~ 10 Ma ago which can be explained by core–mantle interaction.

Unlike to the two ^39^Ar/^40^Ar ages presented here, the K/Ar ages for the Pliocene Styrian volcanic rocks span a much longer time interval. For instance, two K/Ar ages obtained for Neuhaus volcano (VGP group B) differ from each other (Table 1). Only the younger one overlaps with the new ^39^Ar/^40^Ar age of Klöch, but the intermediate directions suggest that both volcanoes recorded one and the same short-living geomagnetic event. Balogh et al. (1994) already suspected the Neuhaus ages to be overestimated because of excess argon. This is strongly supported by our paleomagnetic and dating results. Another K/Ar age is available for one dike from Königsberg (2.17 ± 0.13 Ma, Tieschen, Seghedi et al. 2004, see Table 1). It is slightly younger, but overlaps with the new age for Klöch volcano within a two-sigma error (95% probability). Hence, all lavas of VGP group B and C could have cooled down in a very short time interval. For the sites Waltra and Stradner Kogel, the low paleointensities (Fig. 13) and the pole positions close the VGP cluster B (Fig. 13) suggest that Stradner Kogel volcano could have been formed shortly before or after the geomagnetic excursion. Accordingly, at least 3 or 4 of the Styrian volcanoes may have been formed in a short time interval corresponding to the duration of a geomagnetic cryptochron. It remains difficult to assign the duration of such an event. According to Cande and Kent (1995), the duration is less than 10 to 30 kyr. A recent investigation of the Laschamp excursion (Cooper et al. 2021) used ancient New Zealand kauri trees for investigating atmospheric radiocarbon and revealed a duration of only 1500 years.

## Conclusions

Paleomagnetic directions from 27 sites from 6 volcanic centers and one volcanic complex of the Styrian volcanic field have been obtained and confirm previous results of Pohl and Soffel (1982). All VGPs have reversed polarity. Only 4 are in the range of secular variation, while the others have low reversed VGP latitudes in the South Atlantic. The anomalous directions cannot be explained by local or regional tectonics. Low paleointensities and ^39^Ar/^40^Ar dating support that all intermediate directions were recorded during an unstable field configuration. The Styrian basalts provide further evidence for cryptochron C2r.2r-1 about 2.4 Ma ago.

## Supplementary Information

**Additional file 1: Table S1**: Paleointensity results on specimen level.

**Additional file 2: Table S2**: Raw data of ^39^Ar/^40^Ar- incremental heating experiments.

**Additional file 3: Table S3**: Quality of paleointensity results and outcome of *κ(T)*-curves.

**Additional file 4: Table S4**: Results of hysteresis measurements. Descriptions of data are given within the files.

**Additional file 5: Figure S1**: *κ(T)*-curves (left) and thermal cycling (right). The *κ(T)*-curve type is given (see text). One representative example for each type from each site (cf. Table 1 and S3) is shown.

**Additional file 6: Figure S2:** Directions of NRM (gray, on specimen level) and ChRM (black, on sample level) are plotted together with the mean ChRM (red) and its 95% confidence circle for each site (cf. Table 1) in equal area stereographic net. Open symbols indicate reversed and closed symbols normal directions. Green stars are the mean ChRMs obtained by Pohl and Soffel (1982) from the same rock unit.

**Additional file 7: Figure S3:** Histograms of MDT (top) and MDF (bottom) for the Steinberg site (left) and the Klöch site (right).

**Additional file 8: Figure S4**: Additional information of paleointensity experiments of Fig. 8. NRM decay curve (left) with results of the tail checks, and plots of angular difference of H_L_-pTRM (upper middle), δ CK is the relative check error normalized to the TRM (lower middle), δ t∗ is the normalized tail of pTRM corrected for the angular difference between the applied field and the NRM (upper right) and δ TR is the difference between first and repeated demagnetization (lower right).

**Additional file 9: Figure S5**: ^39^Ar/^40^Ar- incremental heating experiments with degassing spectra (left) and inverse isochron plots (right). a) to d) samples from Klöch volcano and e) to j) samples from Steinberg volcano.

## Data Availability

The directional and paleointensity data associated with this publication are available from https://github.com/PaleoInt/EPS-2021-Styria/.

## Abbreviations

AF: Alternating field
ChRM: Characteristic remanent magnetization
GPTS: Geomagnetic polarity time scale
IRM: Isothermal remanent magnetization
MDT: Median destructive temperature
MDF: Median destructive field
NRM: Natural remanent magnetization
PCA: Principal component analysis
VSM: Vibration sample magnetometer
VGP: Virtual geomagnetic pole
