# Supplementary figures and images for: Intermediate field directions recorded in Pliocene basalts in Styria (Austria): evidence for cryptochron C2r.2r-1
Source: Earth Planets Space. 2021 Oct 3;73(1):182. doi: 10.1186/s40623-021-01518-w (PMC8549934; doi:10.1186/s40623-021-01518-w)

**Figure S1:**

## Altenmarkt

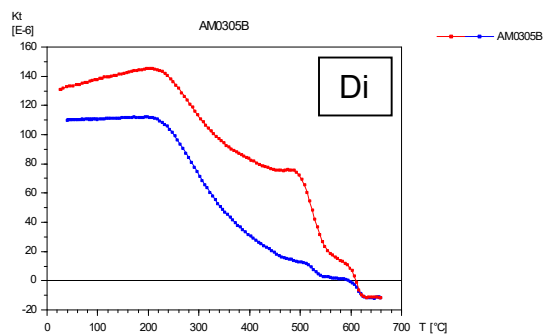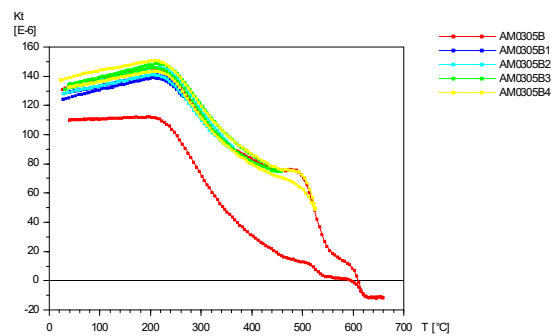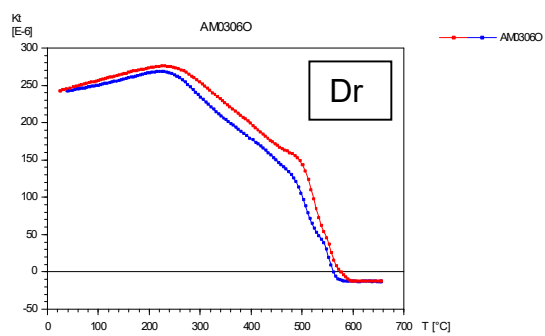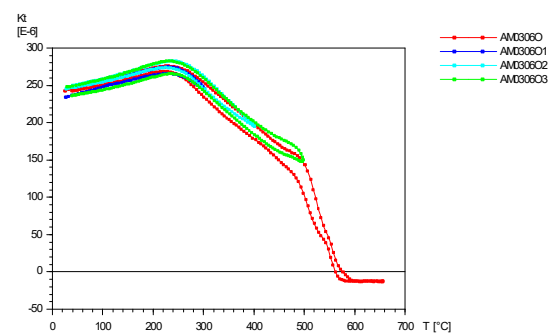

## Klöch

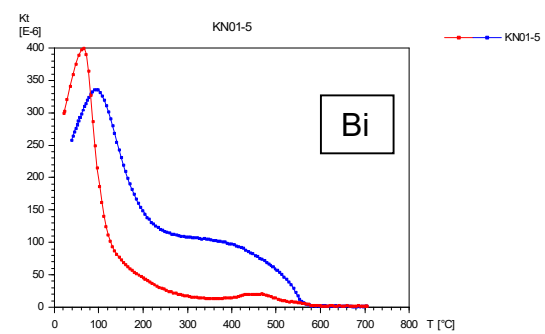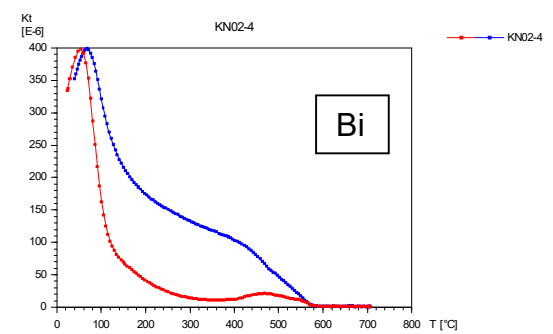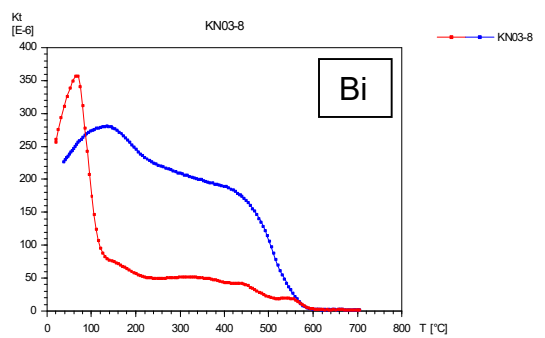

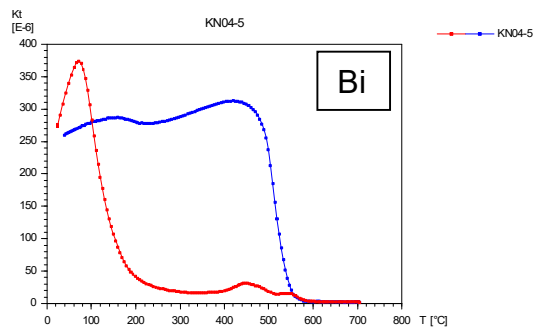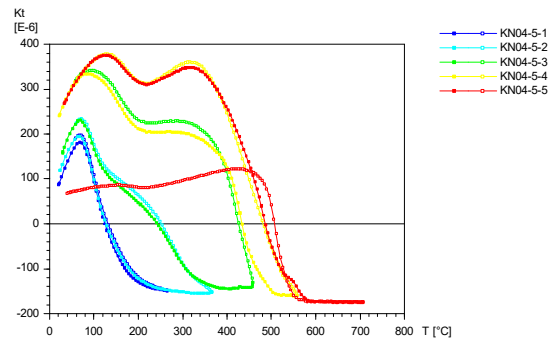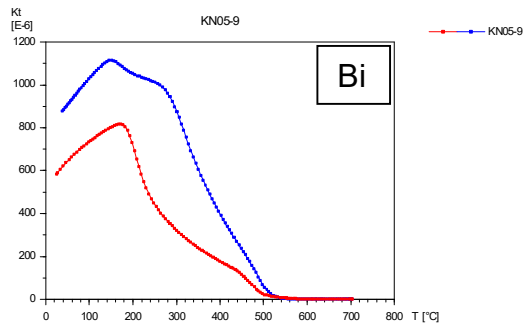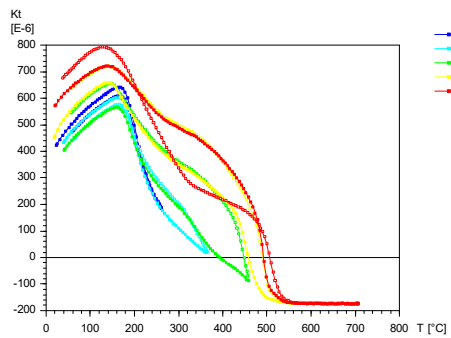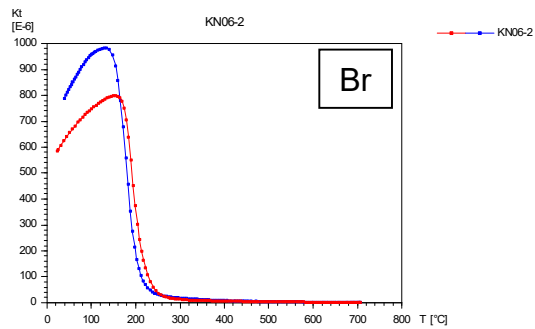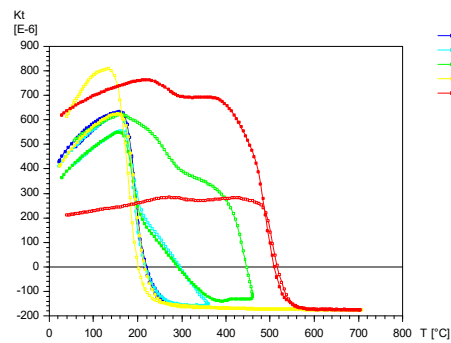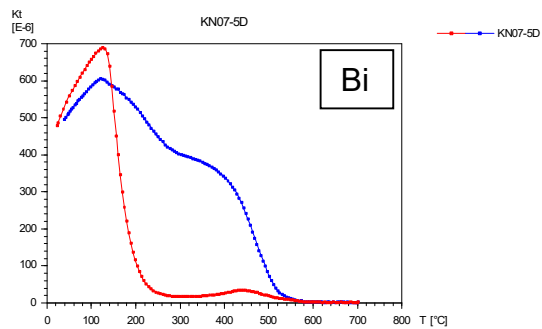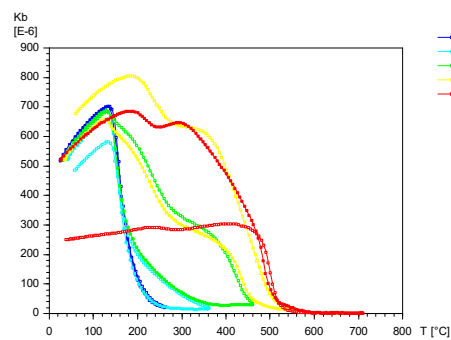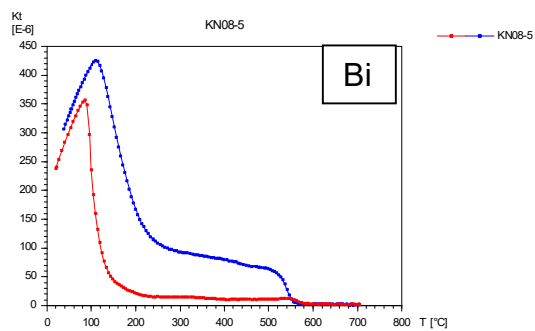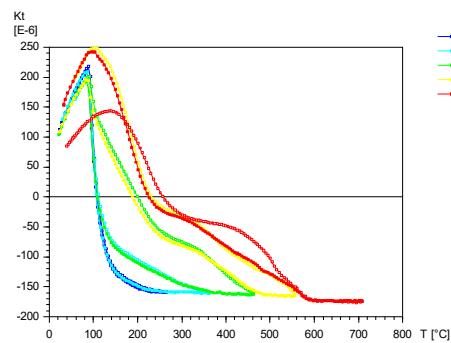

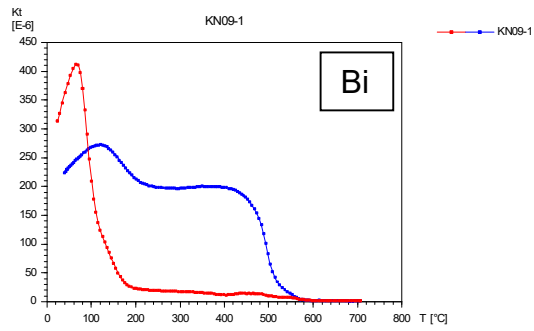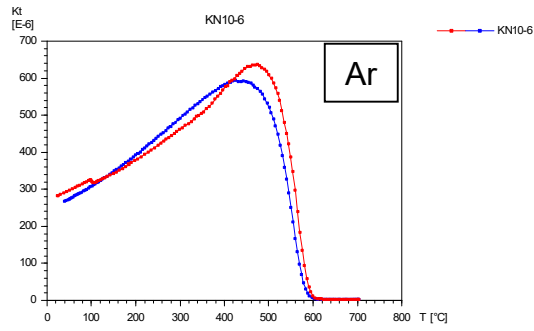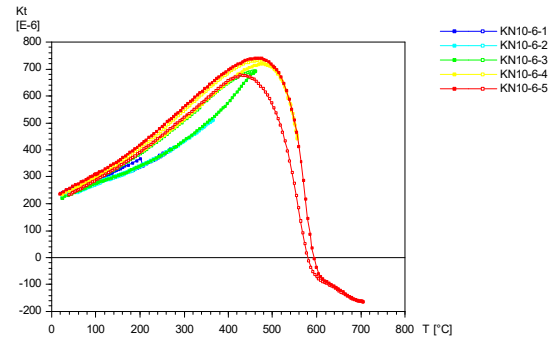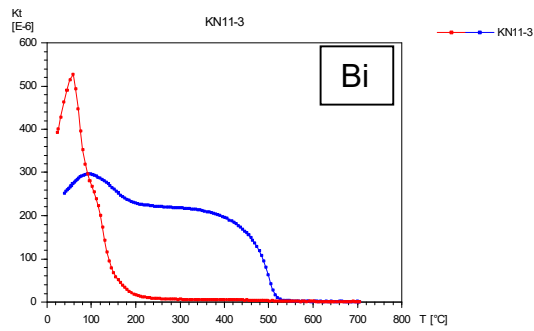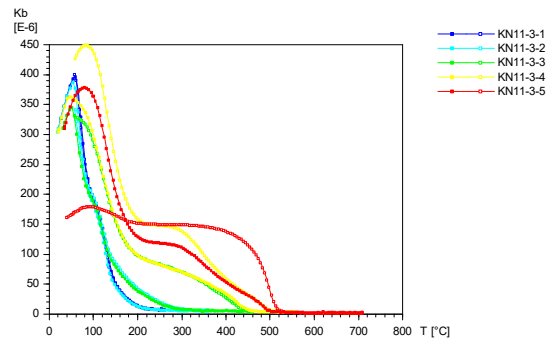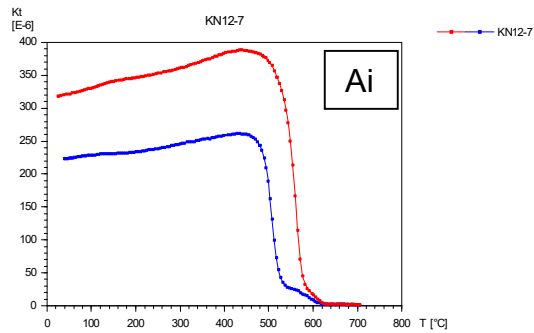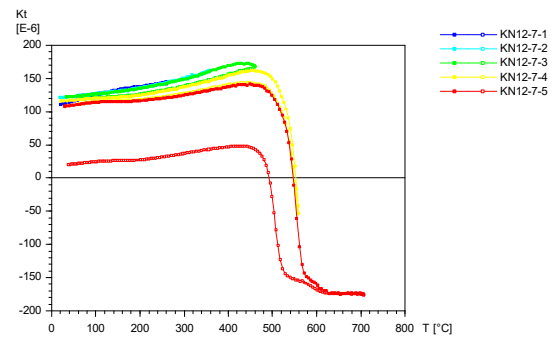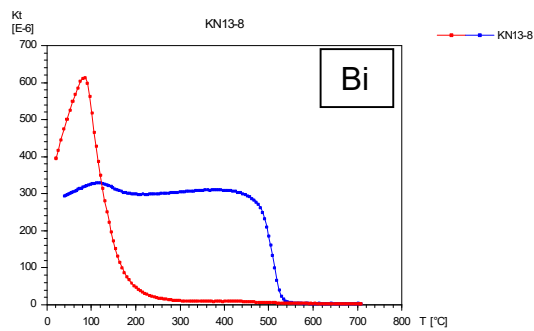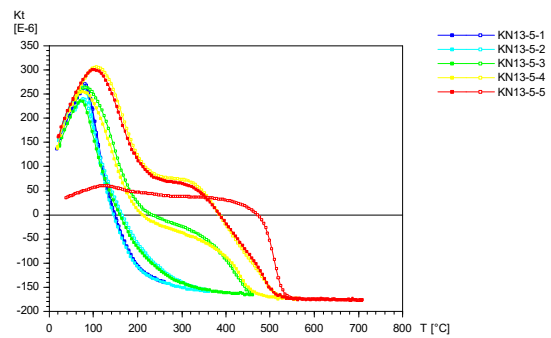

## Zaraberg

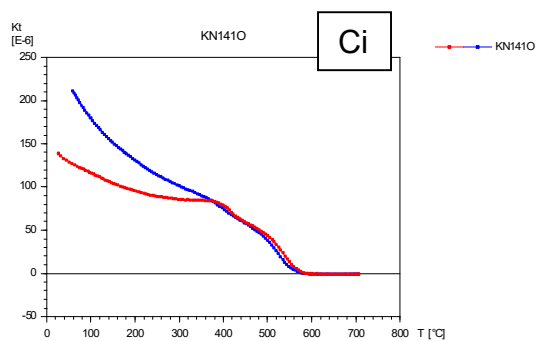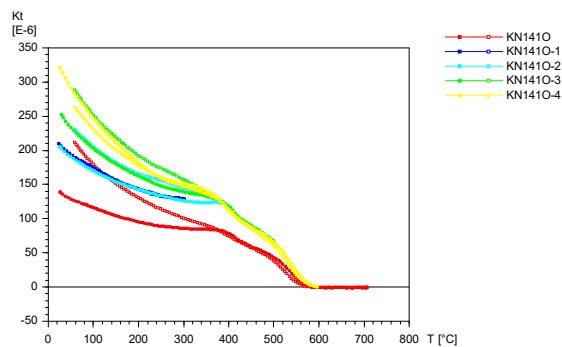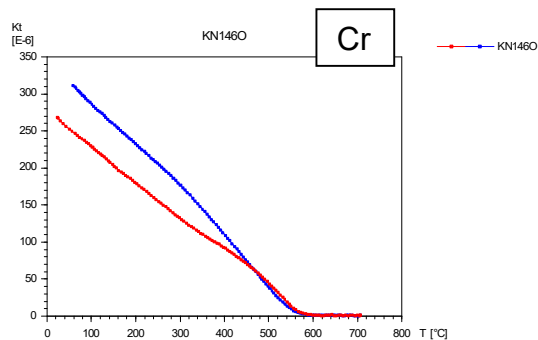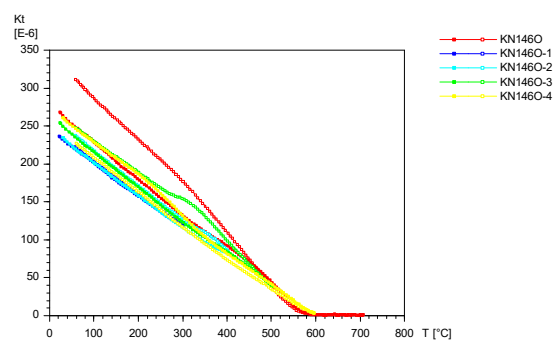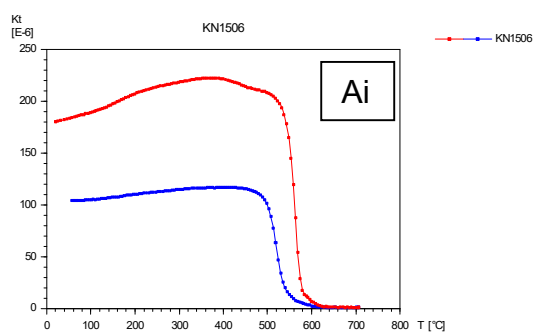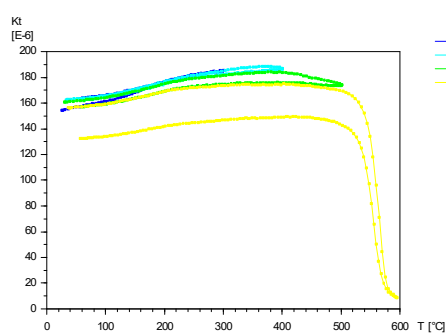

## Königsberg (Tieschen)

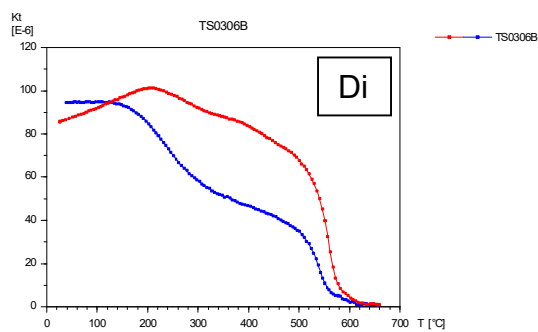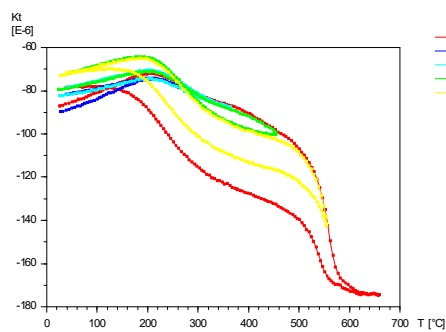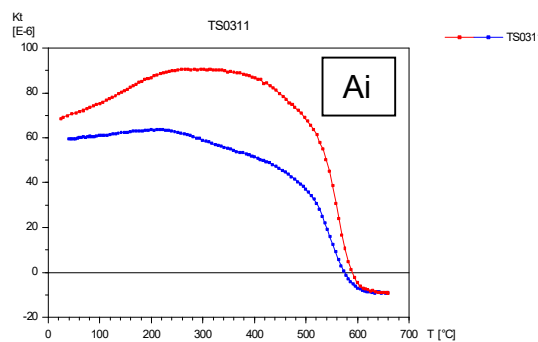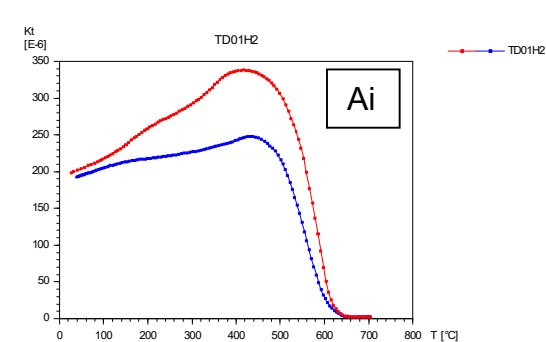

## Neuhaus

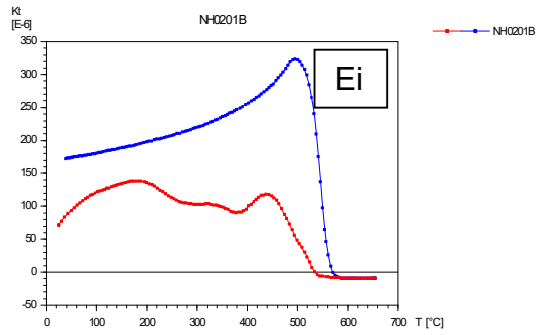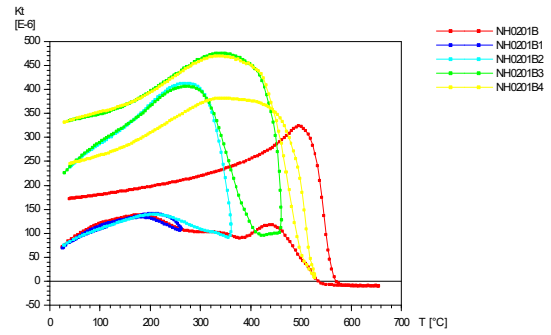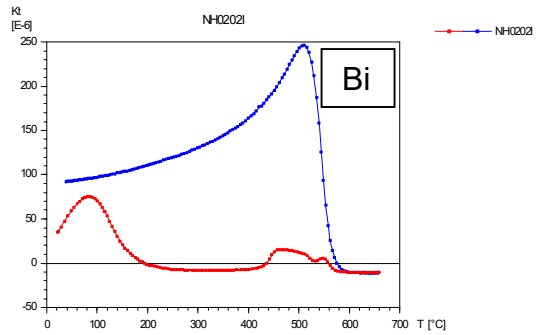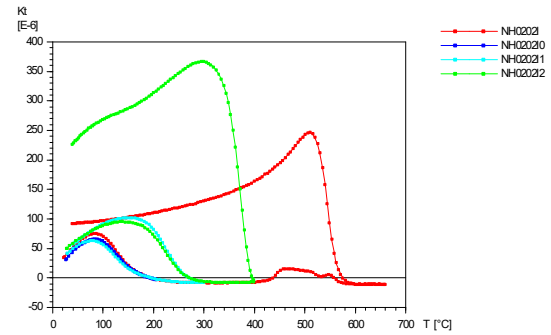

## Steinberg

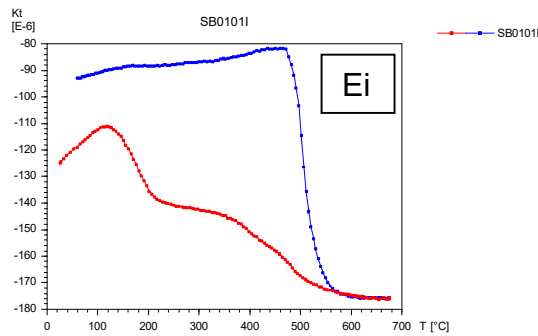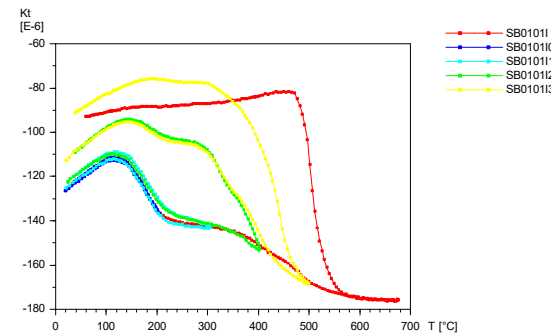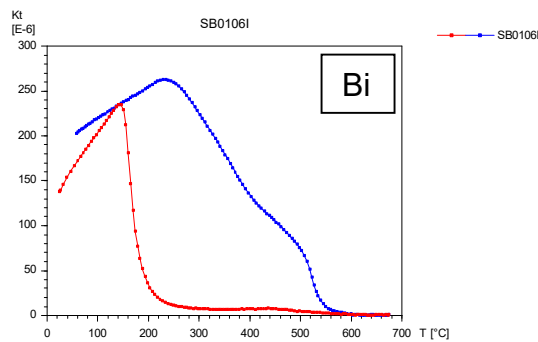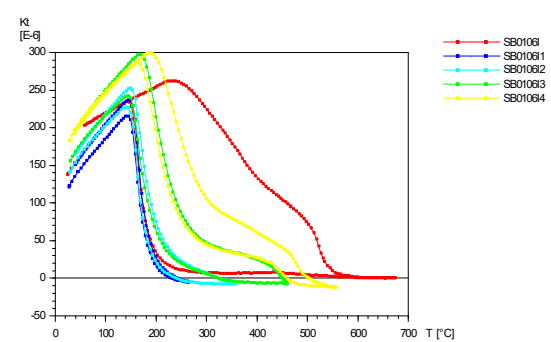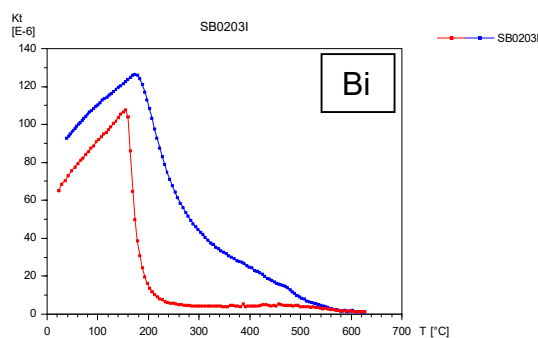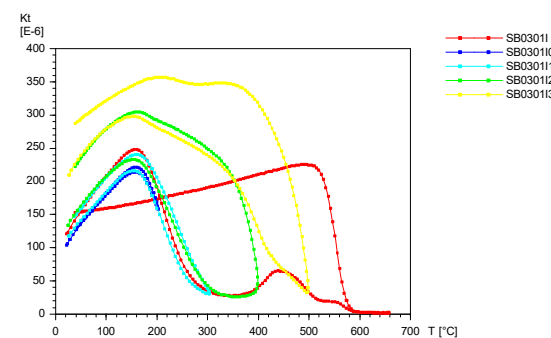

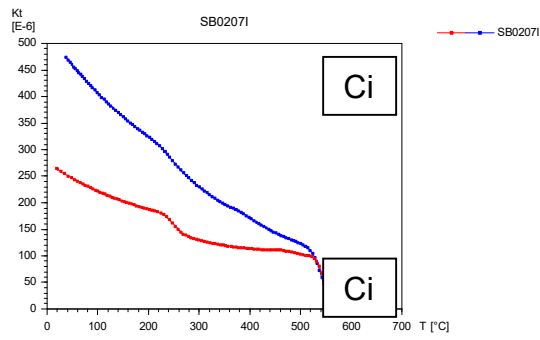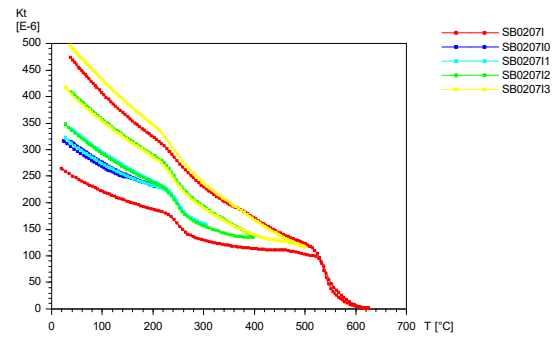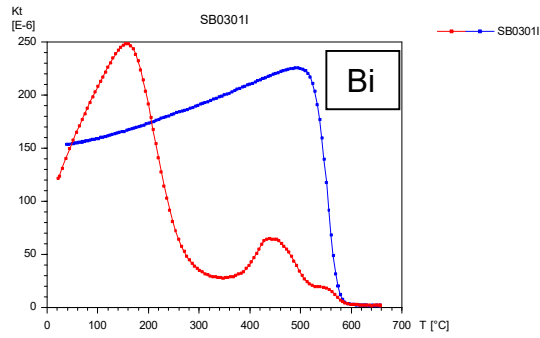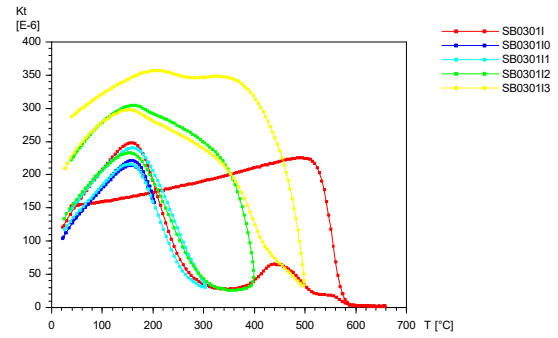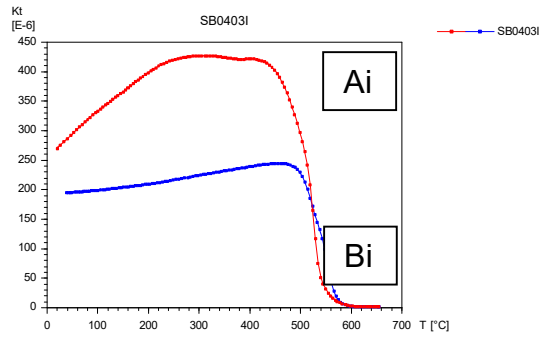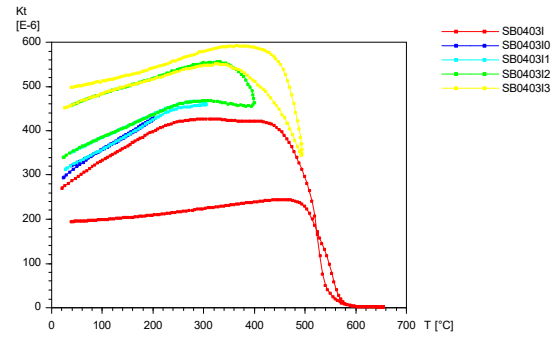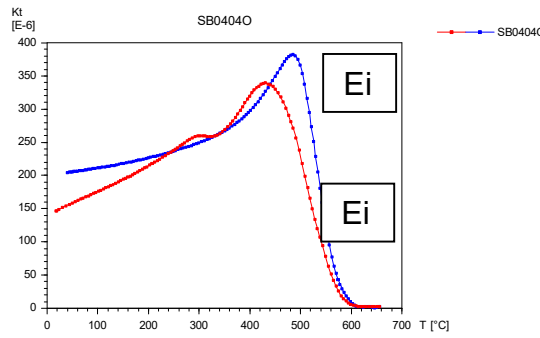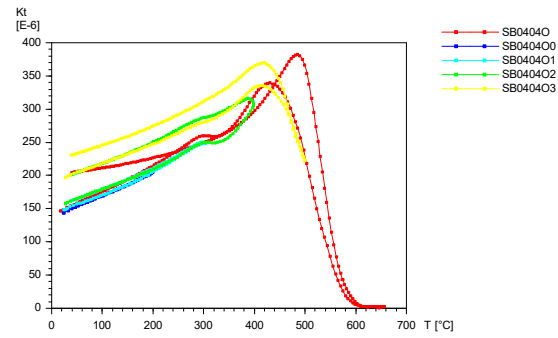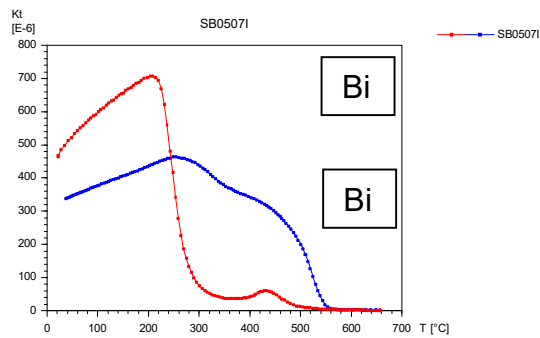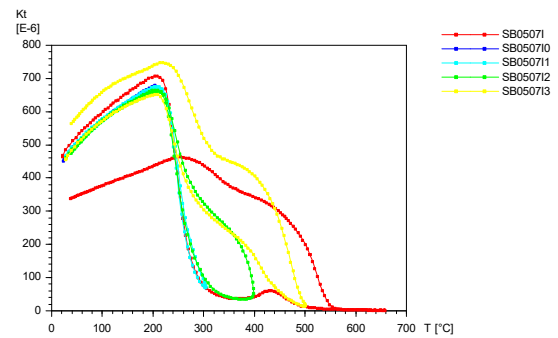

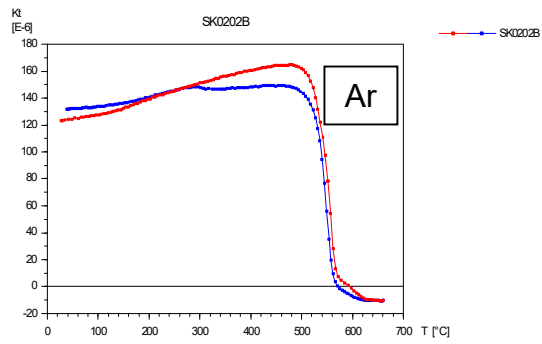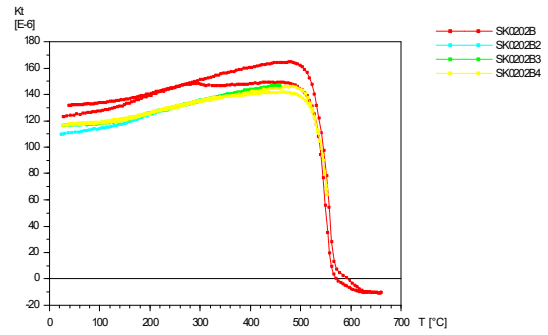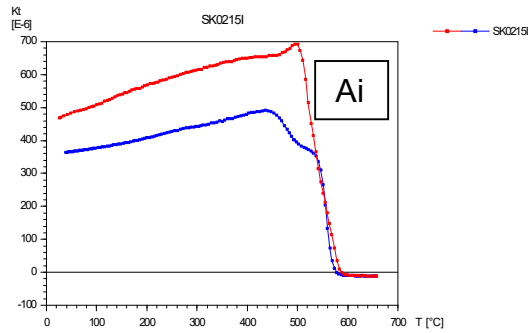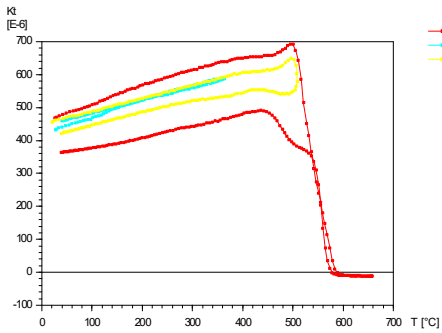

## Stein

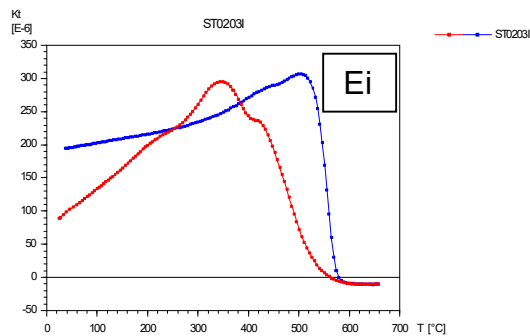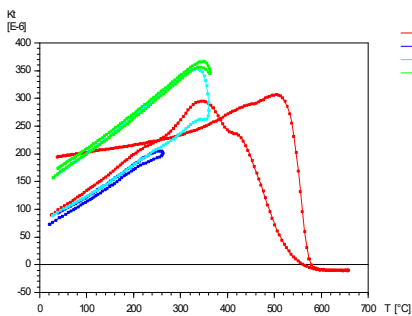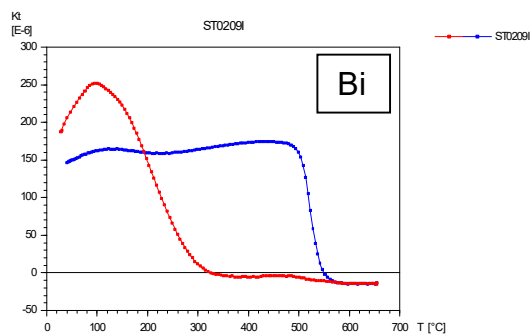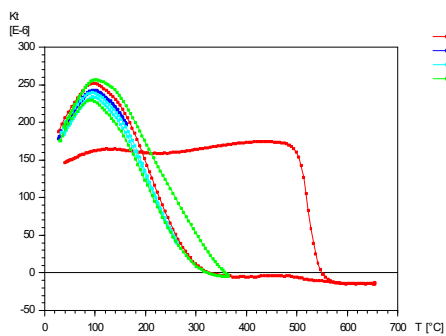

## Waltra

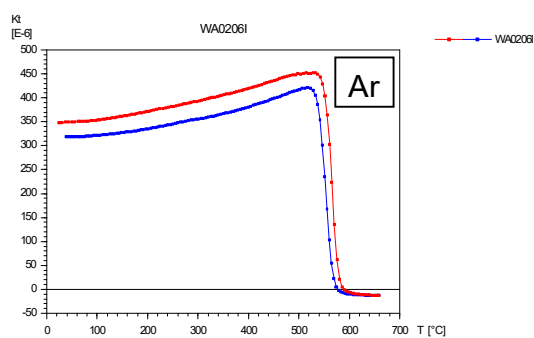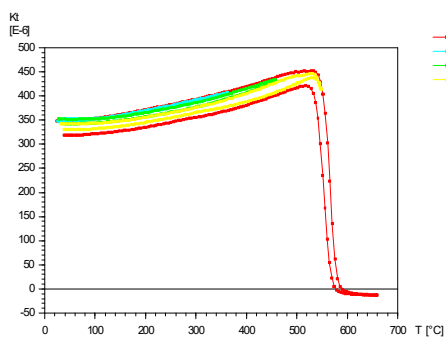

Supplement: Supplementary file 5 — Additional file 5: Figure S1: κ(T)-curves (left) and thermal cycling (right). The κ(T)-curve type is given (see text). One representative example for each type from each site (cf. Table 1 and S3) is shown. [file 40623_2021_1518_MOESM5_ESM.pdf]

Figure S3:

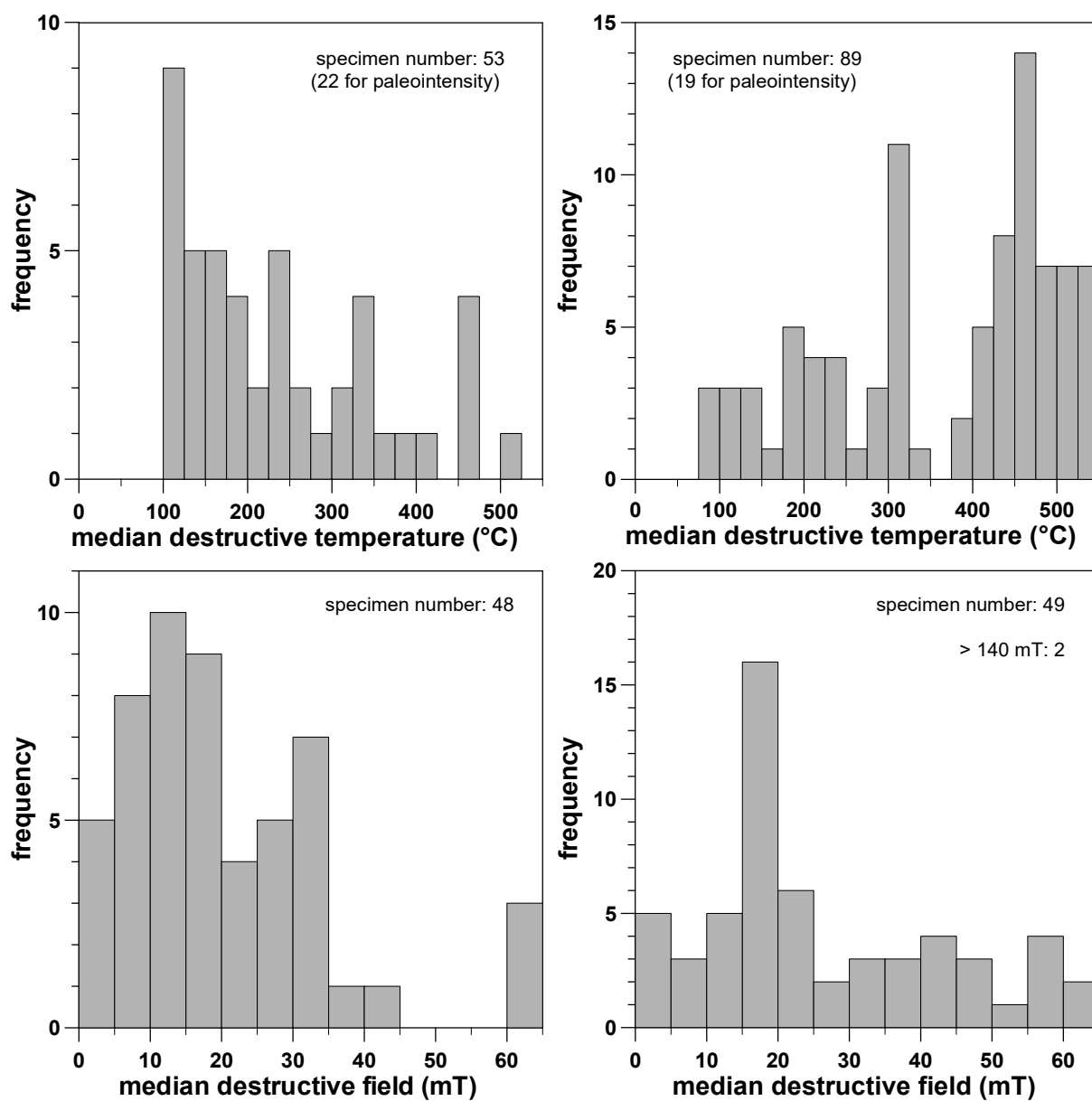

Supplement: Supplementary file 7 — Additional file 7: Figure S3: Histograms of MDT (top) and MDF (bottom) for the Steinberg site (left) and the Klöch site (right). [file 40623_2021_1518_MOESM7_ESM.pdf]
