# Supplementary material for: Intermediate field directions recorded in Pliocene basalts in Styria (Austria): evidence for cryptochron C2r.2r-1
Source: Earth Planets Space. 2021 Oct 3;73(1):182. doi: 10.1186/s40623-021-01518-w (PMC8549934; doi:10.1186/s40623-021-01518-w)
Supplement: Supplementary file 6 — Additional file 6: Figure S2: Directions of NRM (gray, on specimen level) and ChRM (black, on sample level) are plotted together with the mean ChRM (red) and its 95% confidence circle for each site (cf. Table 1) in equal area stereographic net. Open symbols indicate reversed and closed symbols normal directions. Green stars are the mean ChRMs obtained by Pohl and Soffel (1982) from the same rock unit. [file 40623_2021_1518_MOESM6_ESM.pdf]

Figure S2:

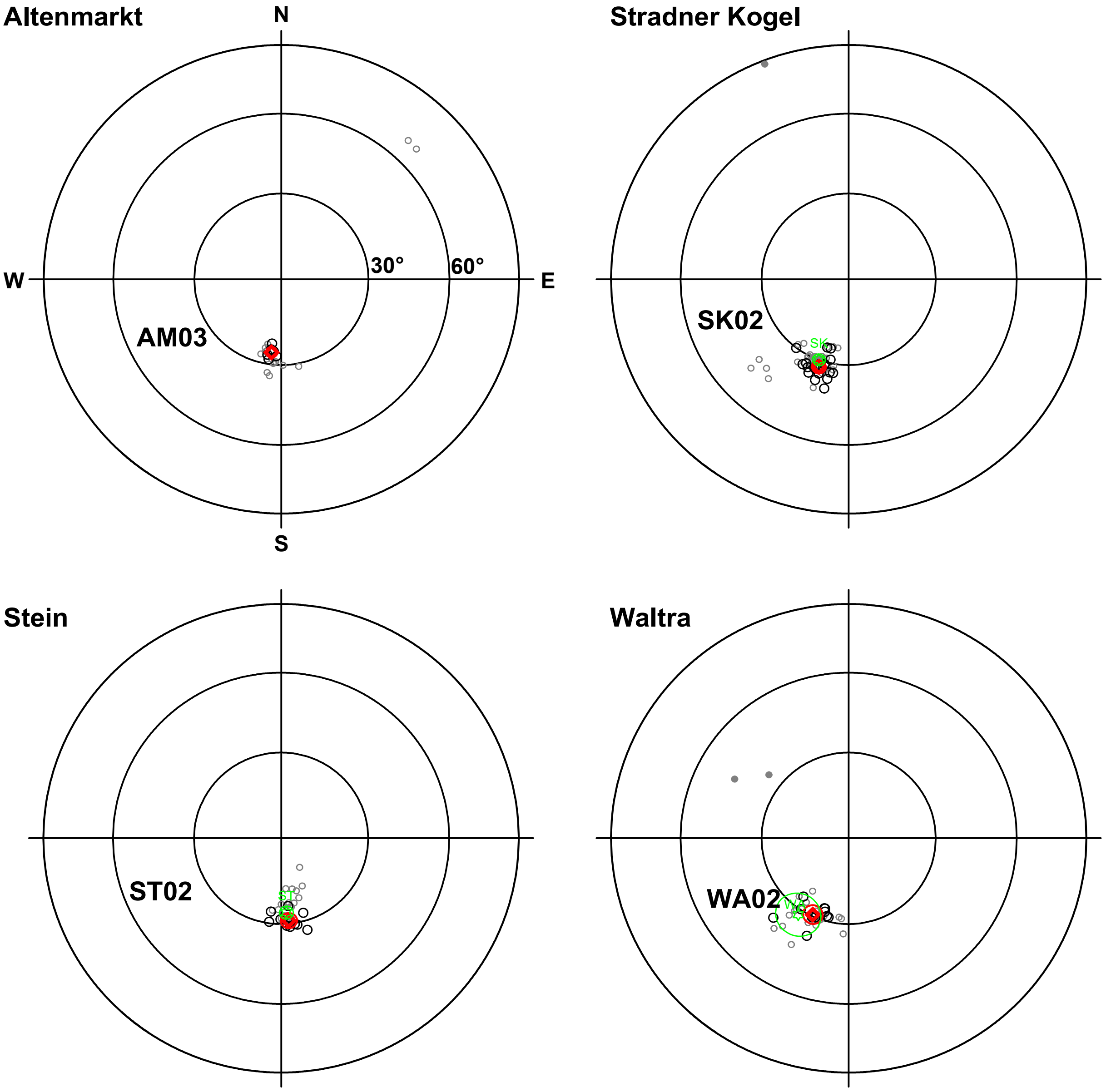
Altenmarkt
N
W
E
S
30°
60°
AM03
Stradner Kogel
SK02
Stein
ST02
Waltra
WA02

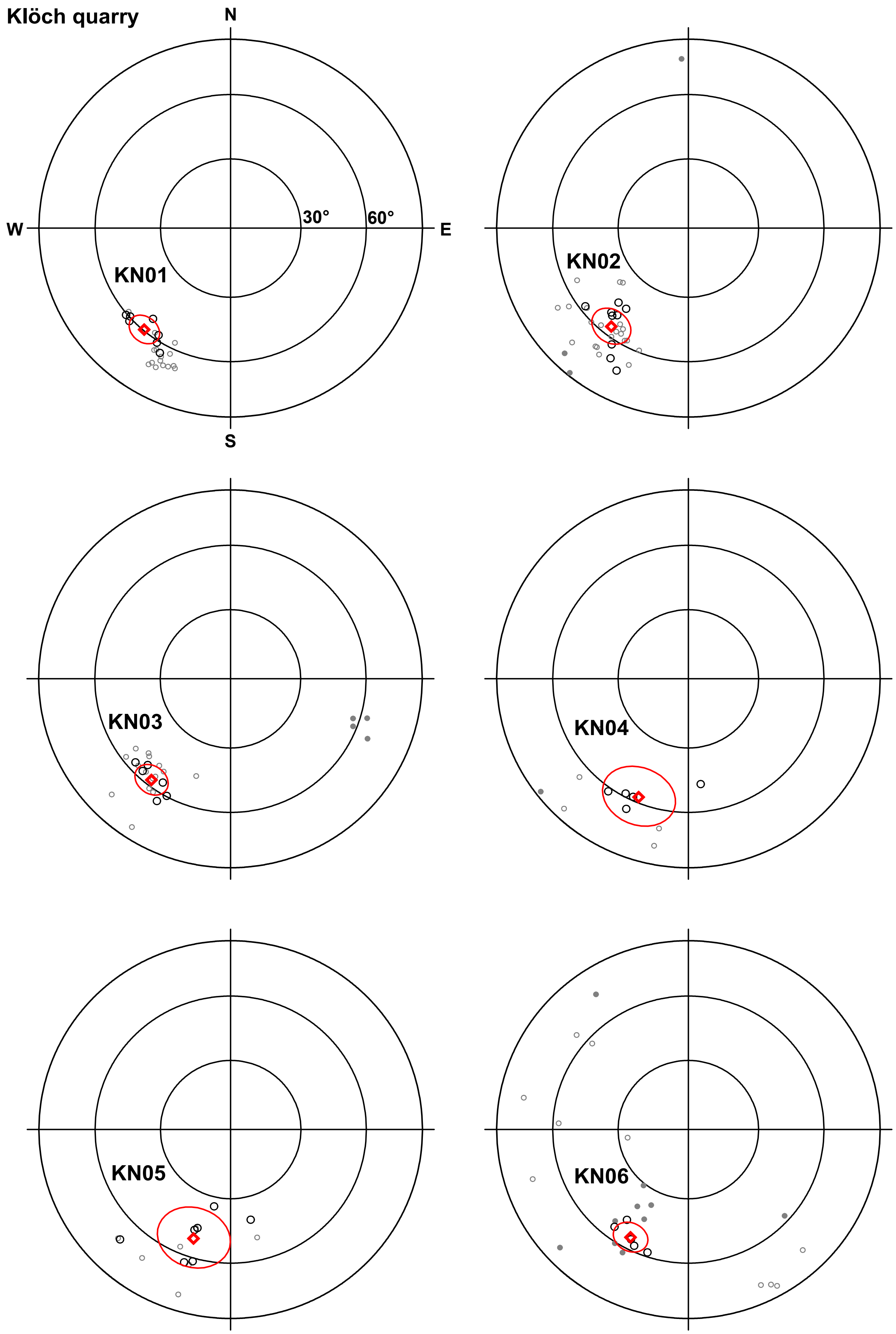
Klöch quarry
N
W
E
S
30°
60°
KN01
KN02
KN03
KN04
KN05
KN06

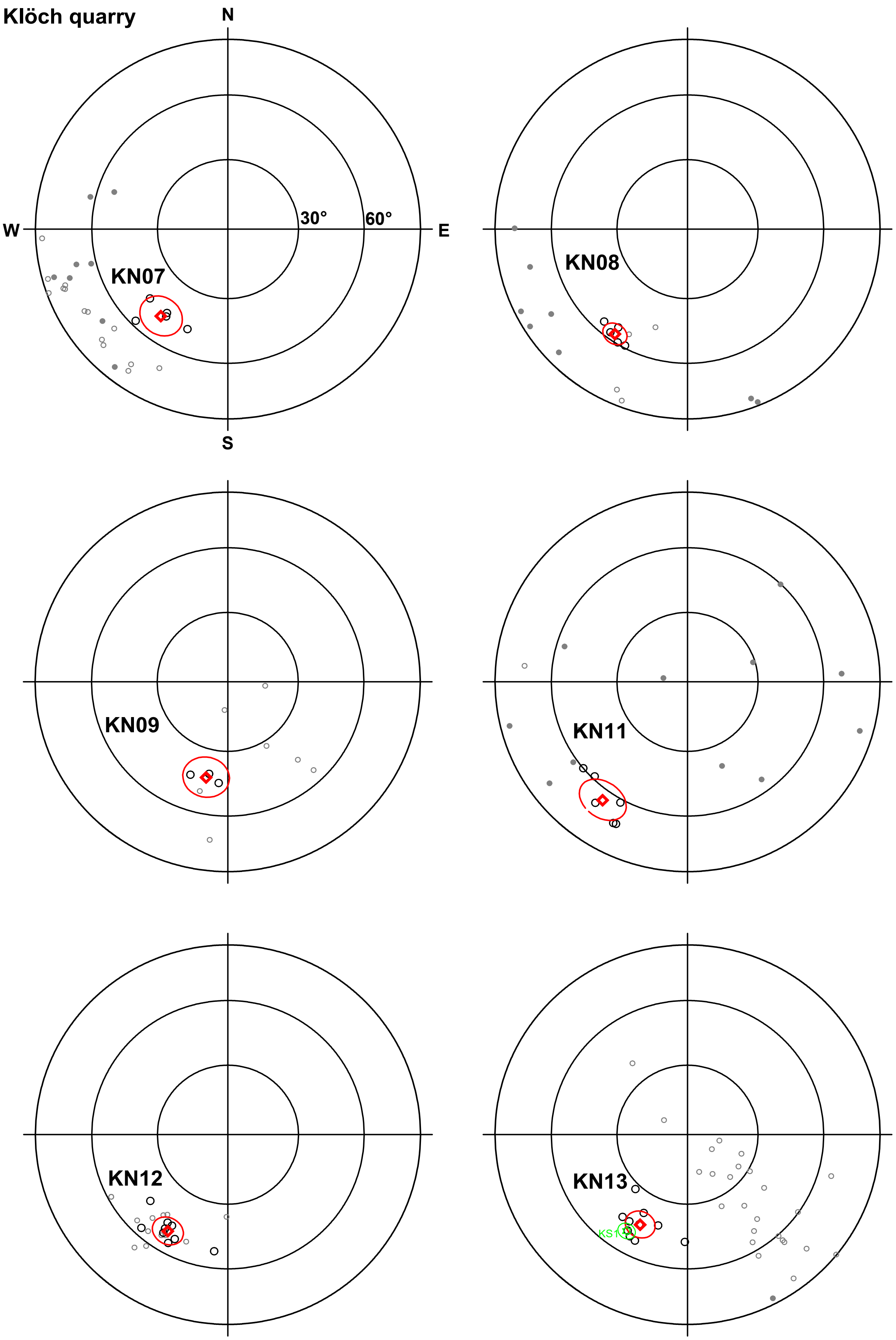
Klöch quarry
N
W
E
S
30°
60°
KN07
KN08
KN09
KN11
KN12
KN13

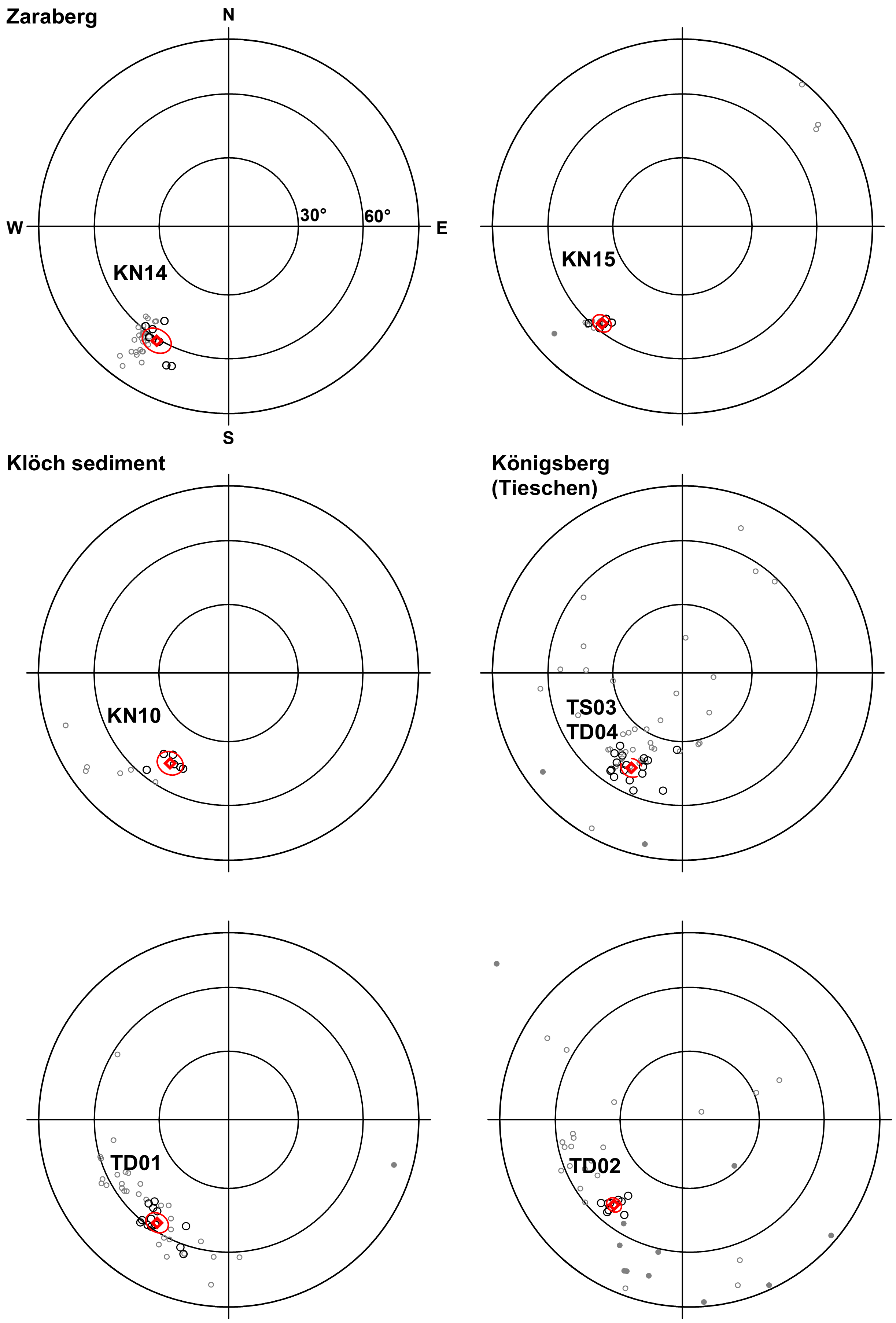
Zaraberg
N
W
E
S
30°
60°
KN14
KN15
Klöch sediment
KN10
Königsberg
(Tieschen)
TS03
TD04
TD01
TD02

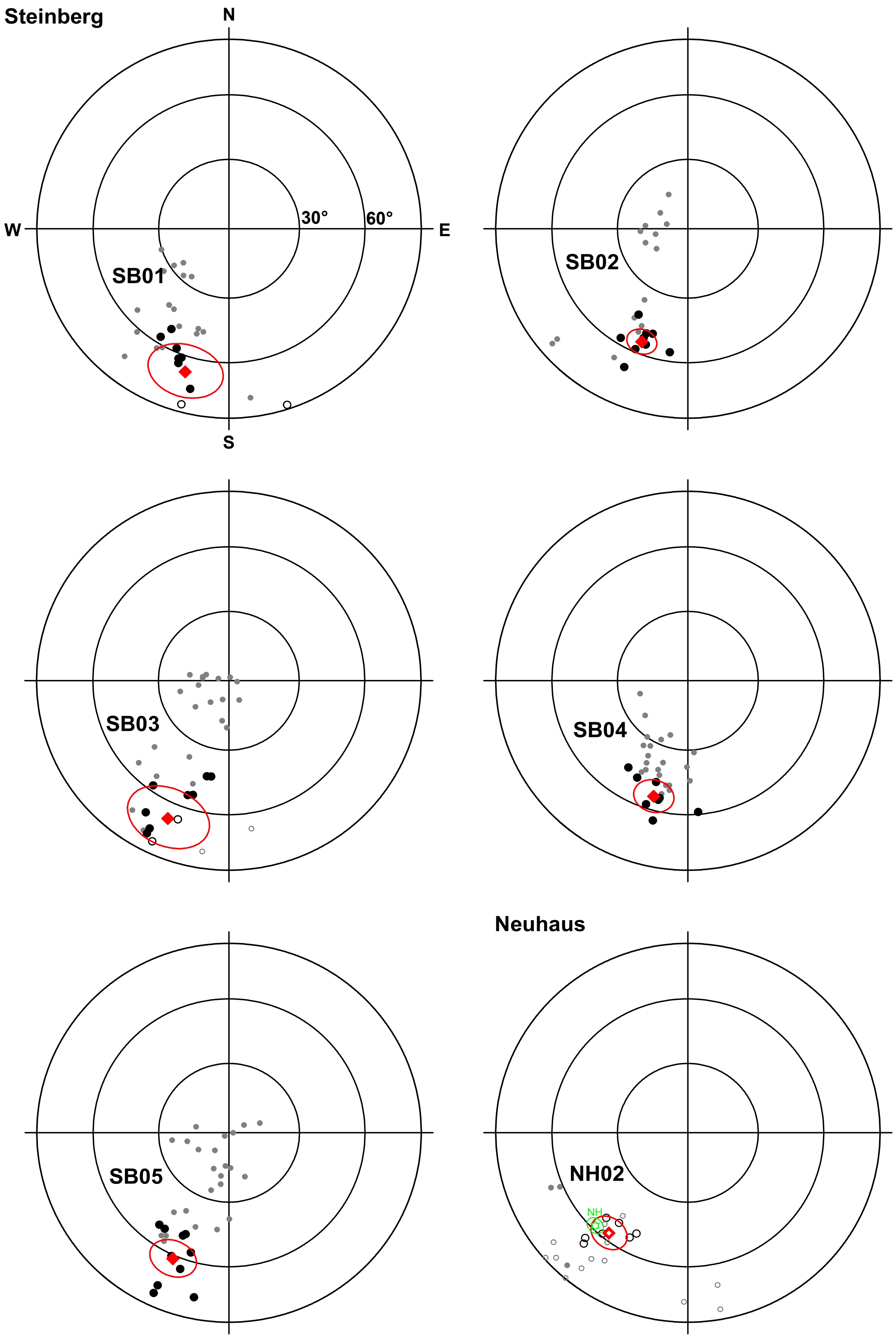
Steinberg
N
30°
60°
W
E
S
SB01
SB02
SB03
SB04
SB05
Neuhaus
NH02
