# Supplementary material for: Intermediate field directions recorded in Pliocene basalts in Styria (Austria): evidence for cryptochron C2r.2r-1
Source: Earth Planets Space. 2021 Oct 3;73(1):182. doi: 10.1186/s40623-021-01518-w (PMC8549934; doi:10.1186/s40623-021-01518-w)

Figure S4:

a) Strict

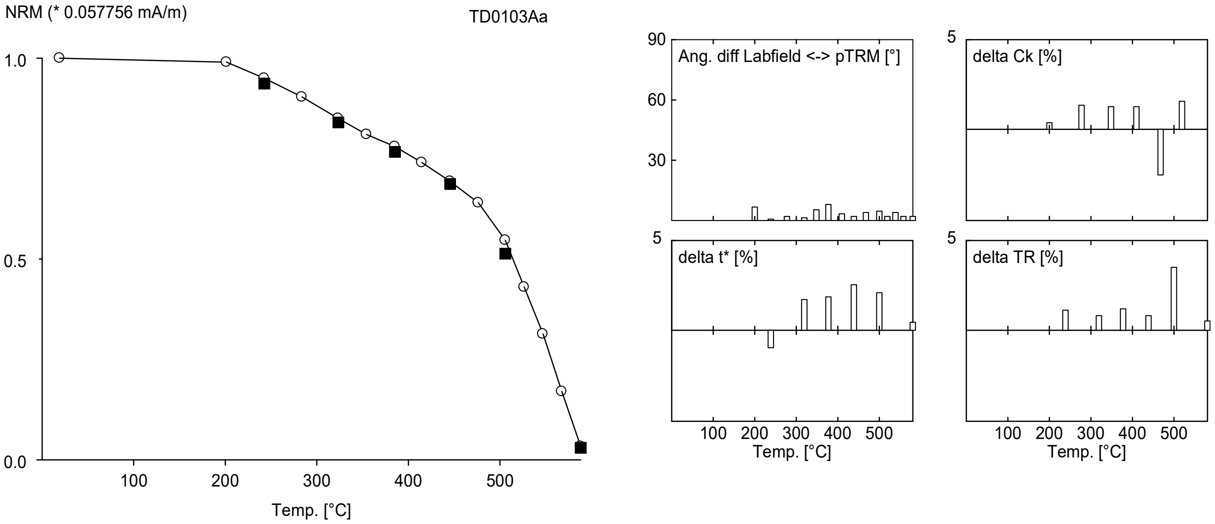

b) Moderate

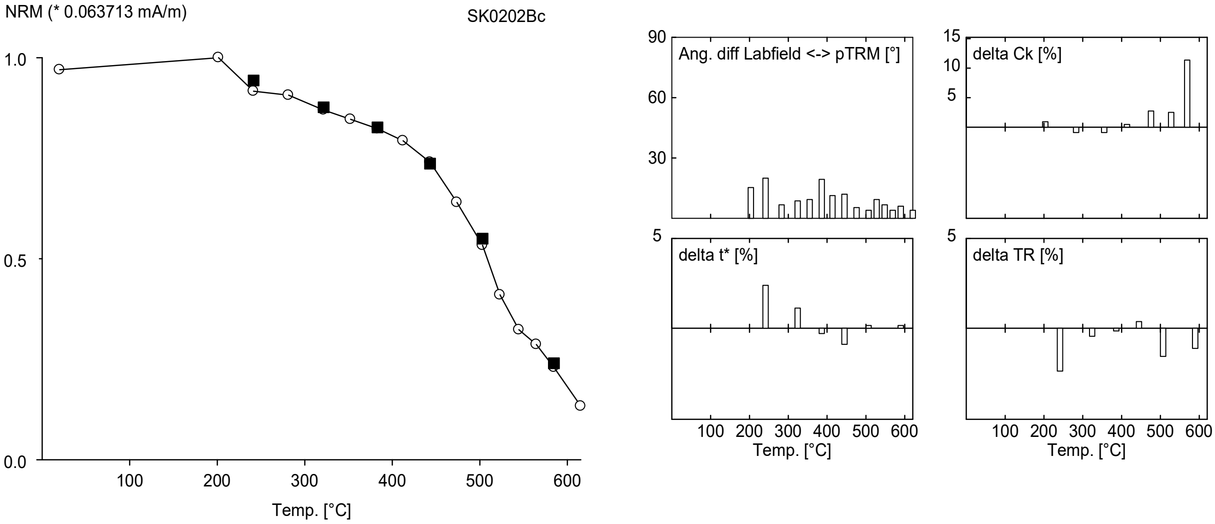

c) Moderate

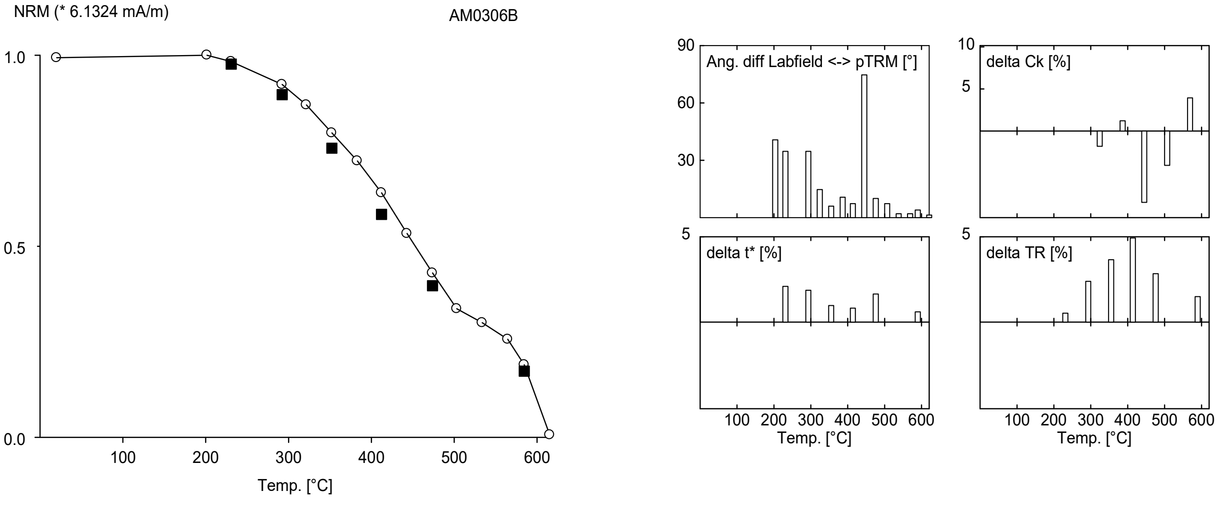

Figure S4 continued:

d) Weak

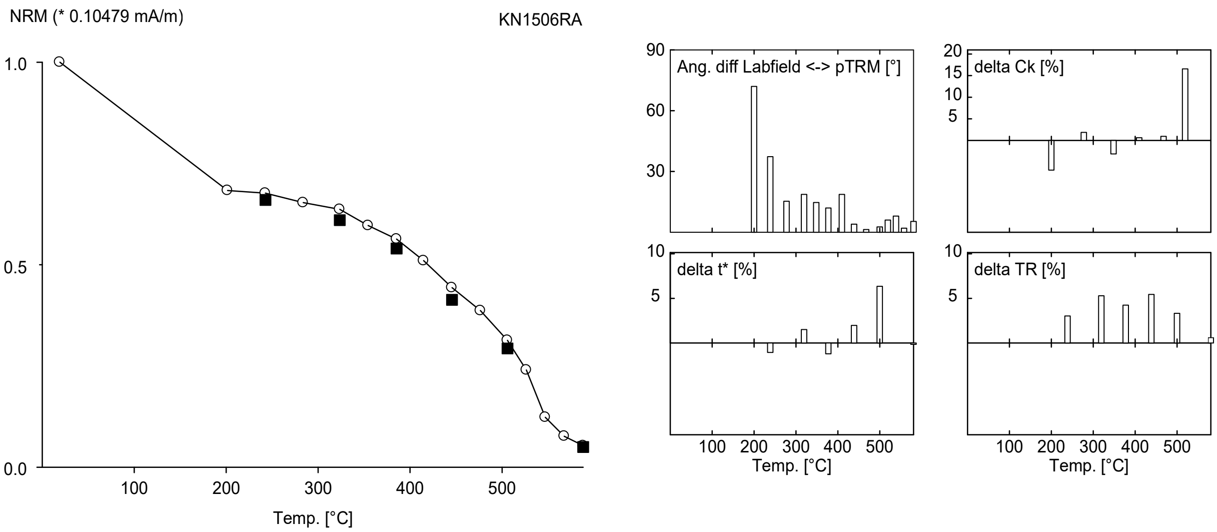

e) Weak

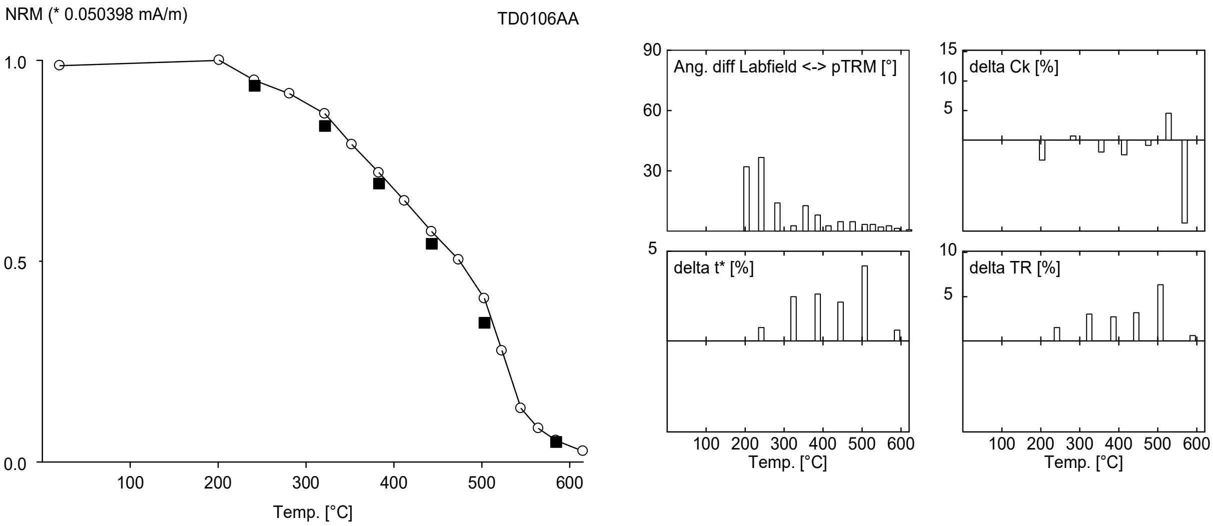

f) failed

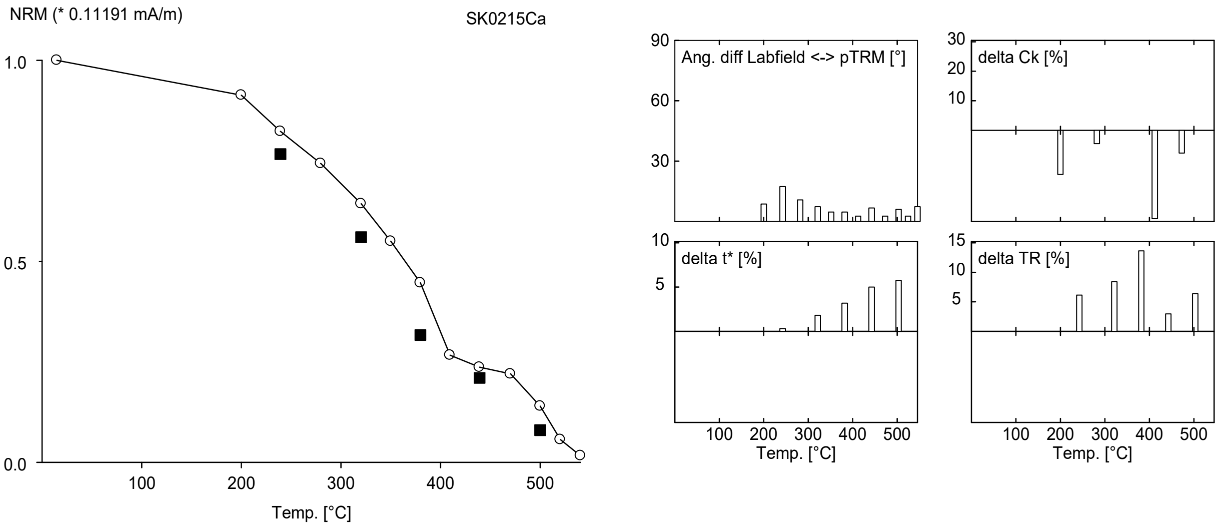

Supplement: Supplementary file 8 — Additional file 8: Figure S4: Additional information of paleointensity experiments of Fig. 8. NRM decay curve (left) with results of the tail checks, and plots of angular difference of HL-pTRM (upper middle), δ CK is the relative check error normalized to the TRM (lower middle), δ t∗ is the normalized tail of pTRM corrected for the angular difference between the applied field and the NRM (upper right) and δ TR is the difference between first and repeated demagnetization (lower right). [file 40623_2021_1518_MOESM8_ESM.pdf]
