# Supplementary material for: Intermediate field directions recorded in Pliocene basalts in Styria (Austria): evidence for cryptochron C2r.2r-1
Source: Earth Planets Space. 2021 Oct 3;73(1):182. doi: 10.1186/s40623-021-01518-w (PMC8549934; doi:10.1186/s40623-021-01518-w)

Figure S5:

a) KN07-5D, groundmass

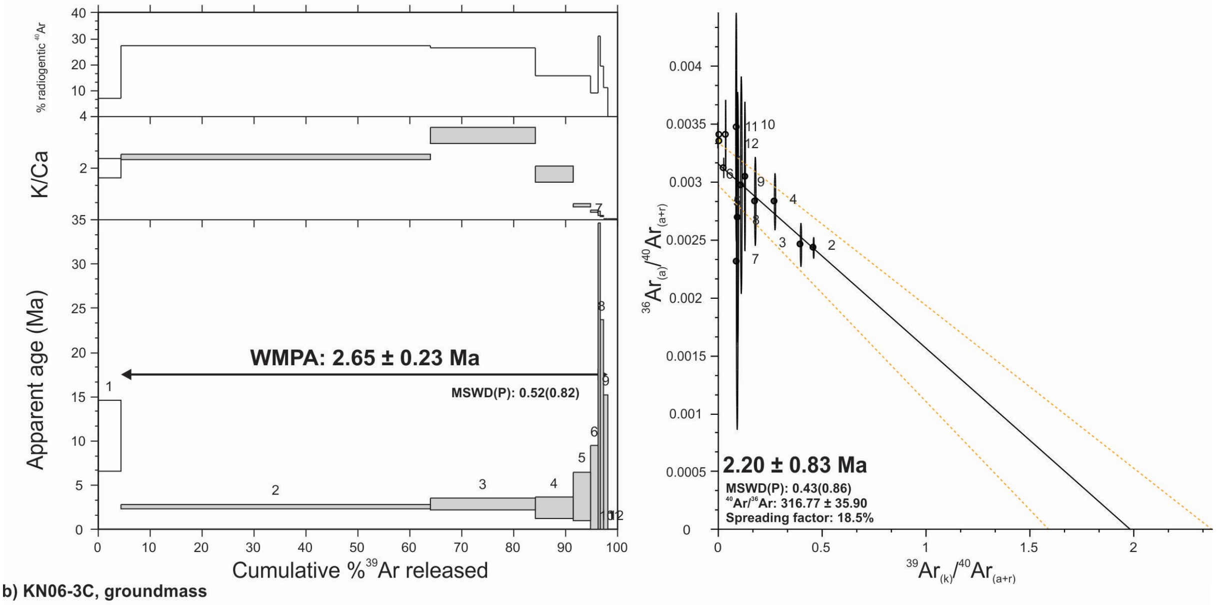

b) KN06-3C, groundmass

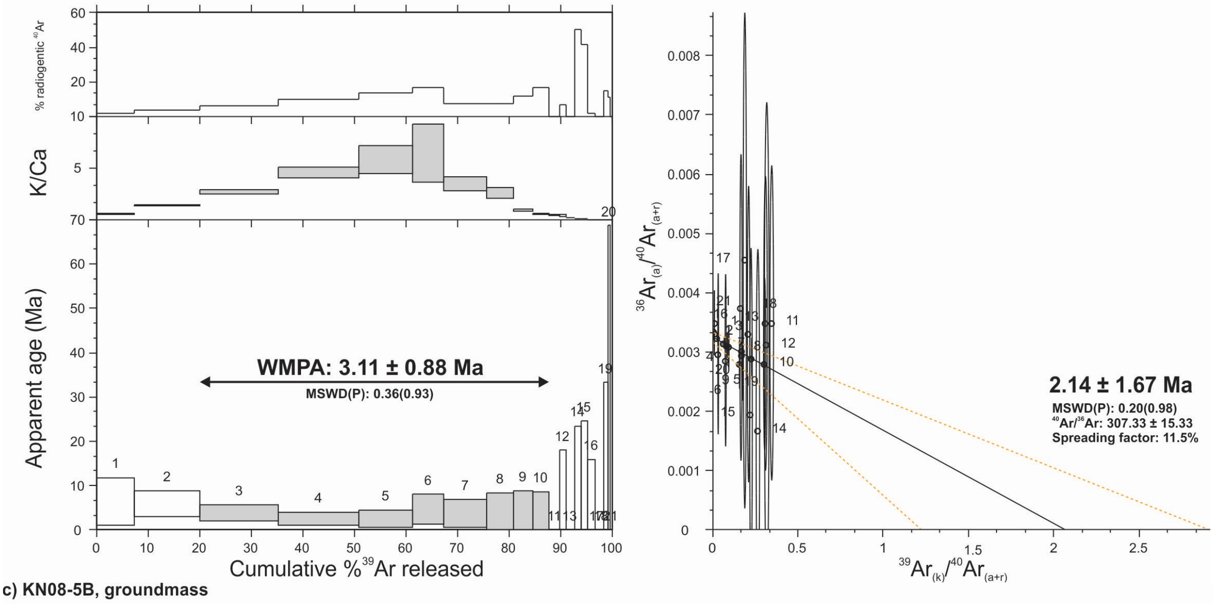

c) KN08-5B, groundmass

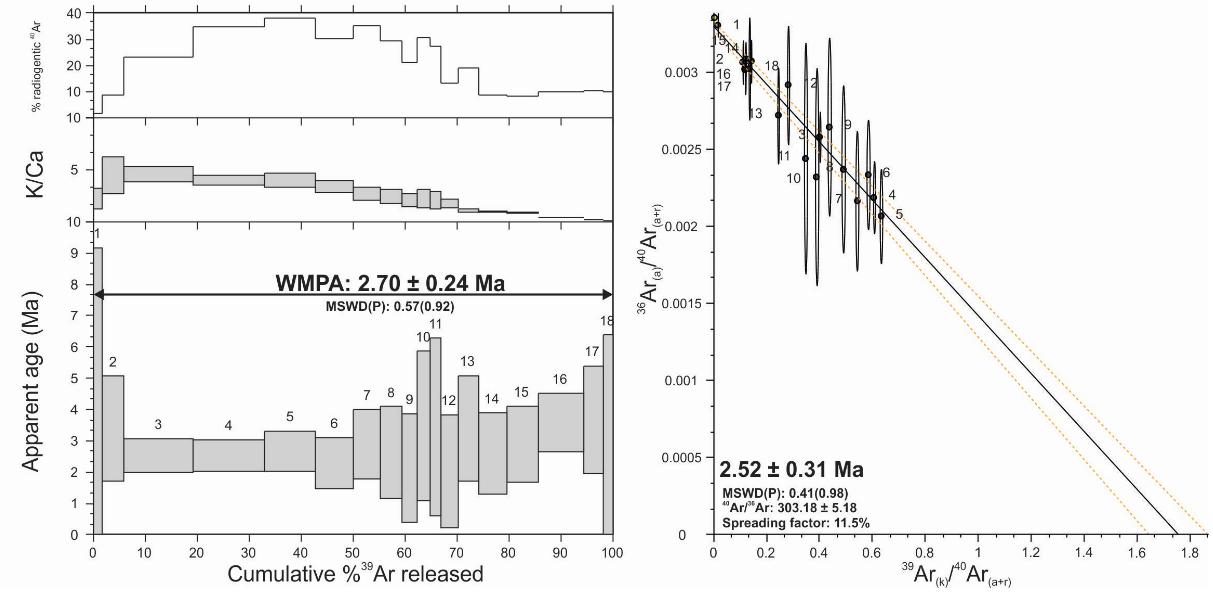

d) KN01-2B, groundmass

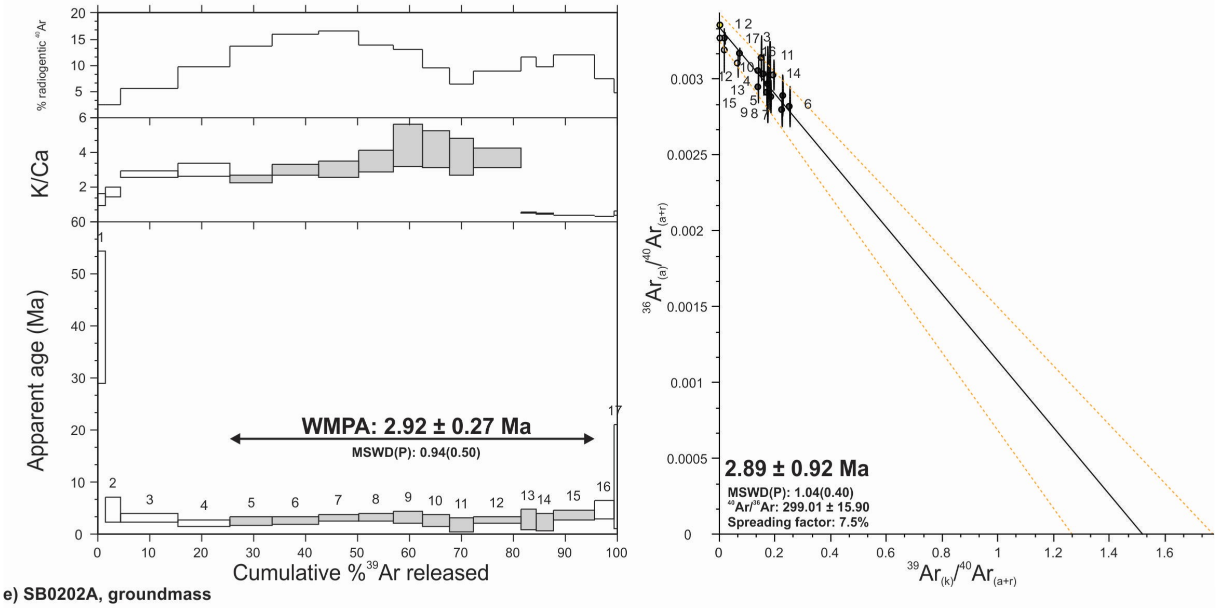

e) SB0202A, groundmass

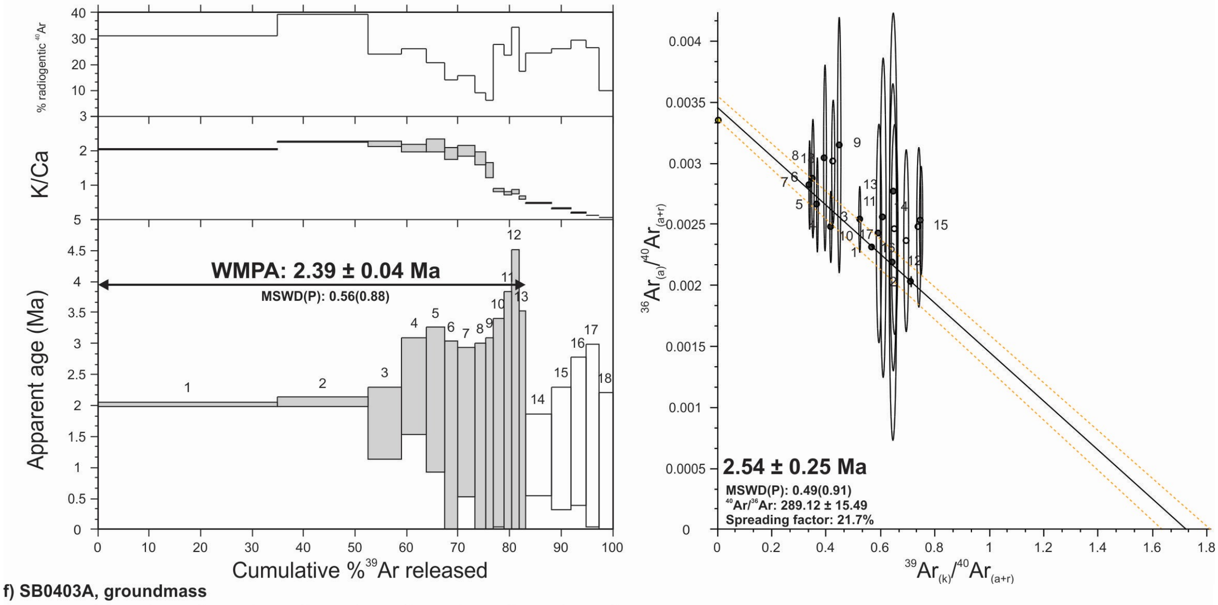

f) SB0403A, groundmass

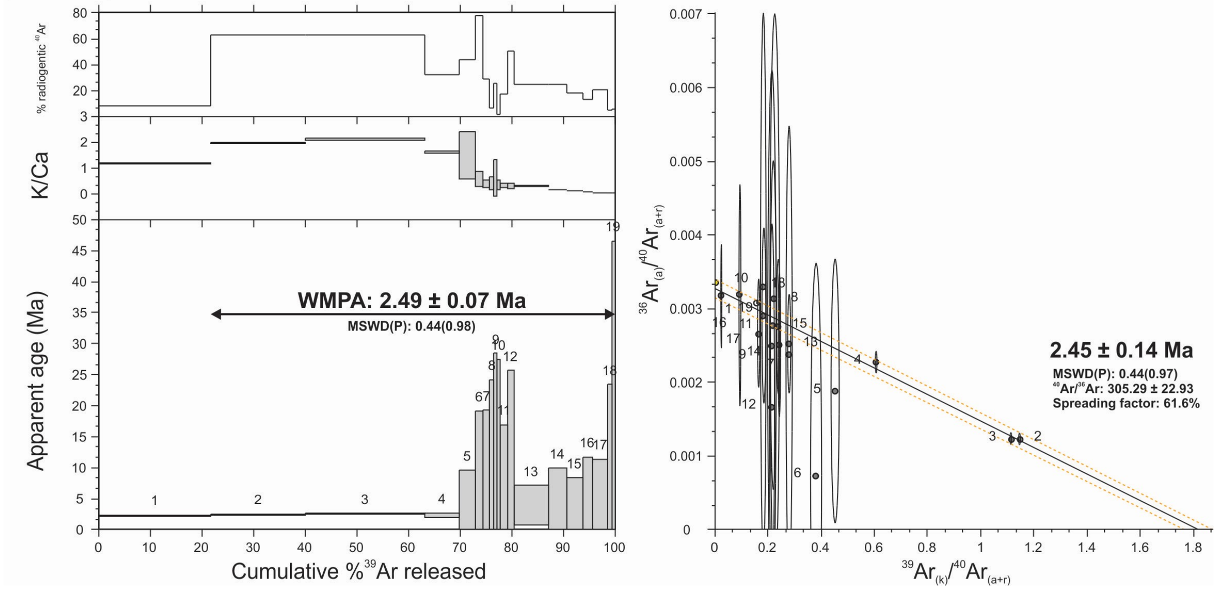

g) SB0402A, groundmass

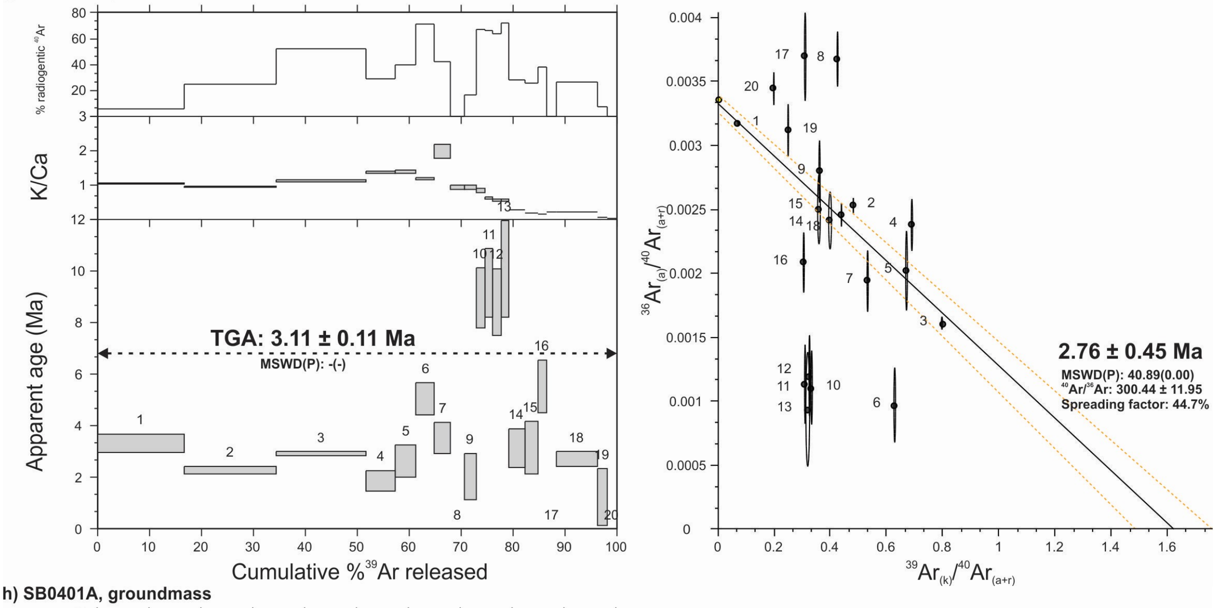

h) SB0401A, groundmass

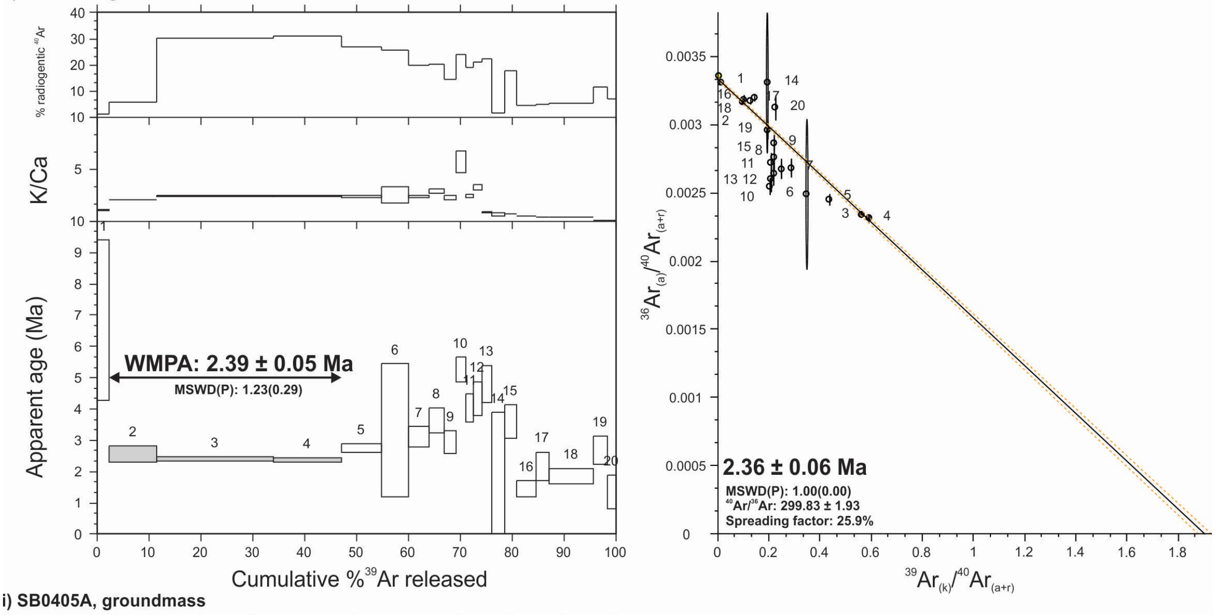

i) SB0405A, groundmass

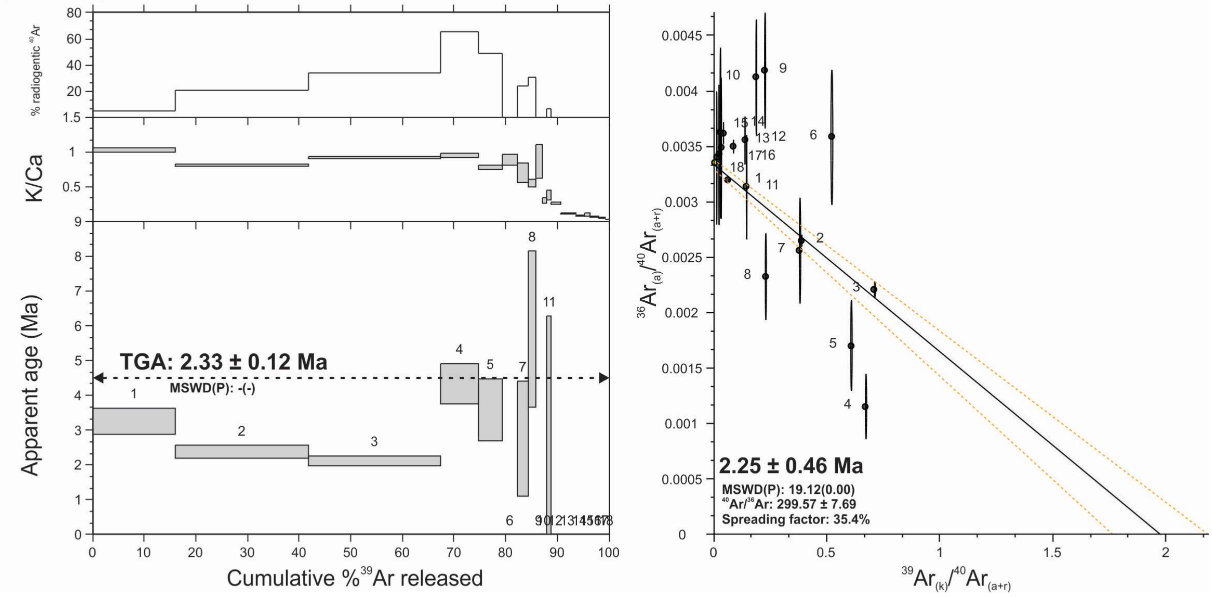

j) SB0404A, groundmass

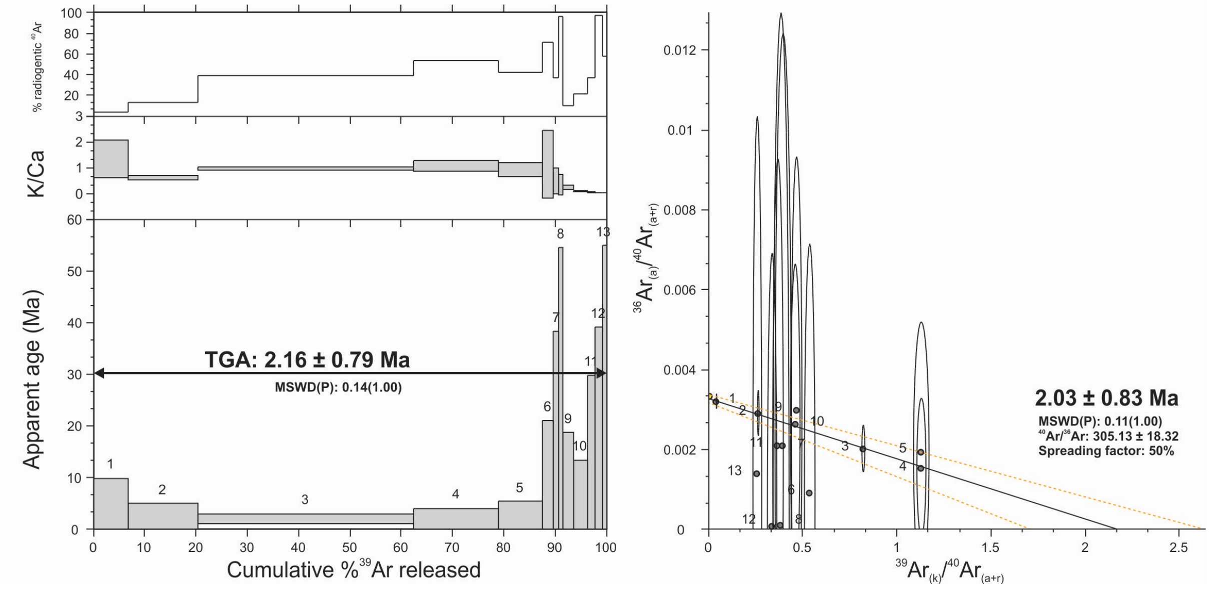

Supplement: Supplementary file 9 — Additional file 9: Figure S5: 39Ar/40Ar- incremental heating experiments with degassing spectra (left) and inverse isochron plots (right). a) to d) samples from Klöch volcano and e) to j) samples from Steinberg volcano. [file 40623_2021_1518_MOESM9_ESM.pdf]
